# A Mixed Protonic–Electronic Conductor Base on the Host–Guest Architecture of 2D Metal–Organic Layers and Inorganic Layers

**DOI:** 10.1002/advs.202205944

**Published:** 2023-04-19

**Authors:** Xing‐Lu He, Bing Shao, Rui‐Kang Huang, Min Dong, Yu‐Qing Tong, Yan Luo, Ting Meng, Fu‐Jie Yang, Zhong Zhang, Jin Huang

**Affiliations:** ^1^ Pharmaceutical College Key Laboratory of Micro‐Nanoscale Bioanalysis and Drug Screening of Guangxi Education Department Guangxi Medical University 530021 Nanning P. R. China; ^2^ School of Chemistry and Pharmaceutical Sciences Guangxi Normal University Guilin 541004 P. R. China; ^3^ Research Institute for Electronic Science Hokkaido University Sapporo 001‐0021 Japan; ^4^ College Chemistry and Chemical Engineering Zhongkai University of Agriculture and Engineering Guangzhou 510275 P. R. China

**Keywords:** 2D, host–guest interaction, intercalation, mixed conductor, metal–organic frameworks

## Abstract

The key to designing and fabricating highly efficient mixed protonic–electronic conductors materials (MPECs) is to integrate the mixed conductive active sites into a single structure, to break through the shortcomings of traditional physical blending. Herein, based on the host–guest interaction, an MPEC is consisted of 2D metal–organic layers and hydrogen‐bonded inorganic layers by the assembly methods of layered intercalation. Noticeably, the 2D intercalated materials (≈1.3 nm) exhibit the proton conductivity and electron conductivity, which are 2.02 × 10^−5^ and 3.84 × 10^−4^ S cm^−1^ at 100 °C and 99% relative humidity, much higher than these of pure 2D metal–organic layers (>>1.0 × 10^−10^ and 2.01×10^−8^ S cm^−1^), respectively. Furthermore, combining accurate structural information and theoretical calculations reveals that the inserted hydrogen‐bonded inorganic layers provide the proton source and a networks of hydrogen−bonds leading to efficient proton transport, meanwhile reducing the bandgap of hybrid architecture and increasing the band electron delocalization of the metal–organic layer to greatly elevate the electron transport of intrinsic 2D metal–organic frameworks.

## Graphical abstract

The guest particles of inorganic ions and/or molecules insert into the interlamination of neutral host framework 2D MOFs, manufacturing the host–guest architecture of 2D metal–organic layers and hydrogen‐bonded inorganic layers. The intercalated layer provides the proton source and reduces the bandgap of hybrid architecture, which leads to the high‐efficiency mixed protonic–electronic transport.

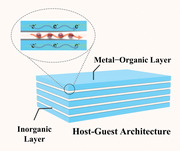

## 1 Introduction

Mixed protonic–electronic conductors (MPECs) possess a wide range of technical applications in devices that rely on mixed ion and electron transport, such as fuel cells, supercapacitors, sensors, flexible electrochromic devices, etc.^[^ ^1^ ^]^ Typically, the MPECs mainly involve conductive polymer composites, ceramics, and ABO_3_ perovskite materials.^[^ ^2^ ^]^ However, the materials of these traditional MPECs are limited by the extreme incompatibility of proton and electron conduction,^[^ ^3^ ^]^ the narrow range of working environmental conditions (such as temperature, and humidity, etc.),^[^ ^4^ ^]^ and poor stability to the environment (such as exposure to air, immersion in body fluids, etc.),^[^ ^5^ ^]^ which limit their practical application. Furthermore, these traditional proton–electron dual‐conductive materials are usually prepared by physical blending, which often results in an unclear structure and transmission path with a large degree of phase boundary, resulting in low transmission efficiency.^[^ ^6^ ^]^ Although some MPECs composites (such as graphene oxides and new fashioned perovskite) exhibit excellent properties, their accurately structural information is unclear, resulting in the uncertain structure–property relationship and unable to elucidate the mixed proton and electron conduction mechanism and interaction.^[^ ^7^ ^]^

Metal–organic frameworks (MOFs), also known as porous coordination polymers (PCPs), is a supramolecular compound constructed by coordination bonds between metal ions/clusters as nodes and organic linking units.^[^ ^8^ ^]^ With the high surface area and porosity, abundant metal centers, regulable structure/composition, MOFs have been widely used in the investigation of gas adsorption and separation, catalysis, sensors, and medicine.^[^ ^9^ ^]^ Notably, a growing number of researches have focused on their role as proton or electron conduction materials, whereas these two developments are in parallel.^[^ ^10^ ^]^ According to the survey, there has been a few MOF‐based MPECs due to the design strategies and components.^[^ ^11^ ^]^ The conduction of MOFs originates from the conduction of carrying charges (protons and electrons) in the host frame and/or the conduction of protons and electrons realized by guest carriers (including some small molecules, ions and nanometal particles, etc.) based on the host–guest interaction.^[^ ^12^ ^]^ However, The design of MOF‐based MPECs adopts a single conduction mode, that is, both ion and electron conduction originate from the same host frameworks or guest carrier, which not only leads to low transmission efficiency, but also involves components that interfere with each other.^[^ ^9b^ ^]^ Therefore, the construction of individually functionalized host–guest architectures to integrate the mixed conductive active sites into a single structure, which is an efficient strategy for the preparation of MOF‐derived MPECs.^[^ ^11a–c^ ^]^

Analogous to other 2D materials, the layers of 2D MOFs (metal–organic layers connected by coordination bonds) is interconnected through hydrogen bonds, *π*–*π* stacking, multiple interactions between the wrinkled surface and/or other noncovalent effects are connected together.^[^ ^13^ ^]^ These metal–organic layers may be electrically neutral, positively, or negatively charged, and the interlayer space of which is a network of empty lattice sites, easy for filling by the guests, such as a combination of protons, solvent molecules and counter ions.^[^ ^14^ ^]^ Especially, the interlayer force of 2D MOFs is weak and the interlayer spacing possesses self‐coordination, which not only breaks through the pore nature of traditional 3D MOFs and restricts the species and components of the guests, but also exhibits high mass permeability coefficient.^[^ ^15^ ^]^ It indicates that 2D MOFs may be one of the best candidates for rational design and synthesis of high performance MPECs. Nevertheless, it is still a great challenge to entry guests into the interlamination of 2D MOFs in a controllable operation and determine their accurately structural information.

## 2 Results and Discussion

**Figure 1:**
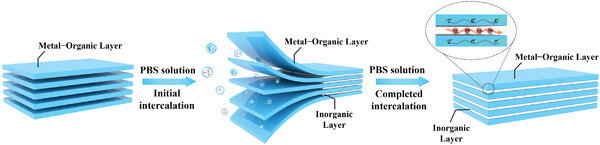
Schematic illustration of the preparation of host–guest architecture of 2D metal–organic layers and ionic layers and mixed protonic–electronic conduction.

The layered intercalation is one of the simplest and most effective methods to introduce guests between 2D materials.^[^ ^16^ ^]^ Based on the interaction/reaction between the host and the guest of 2D materials, the predesigned guests stacking networks structure is inserted into the interlamination of parent layers to obtain desired properties and broaden its application range.^[^ ^17^ ^]^ The controlled preparation of the layer‐like stacking engineering is helpful for the construction of Van der Waals heterostructures (HSs) based on crystal engineering.^[^ ^18^ ^]^ In this work, a neutral 2D MOF with (–Co–O–)_∞_ chairs was constructed with Co^2+^ and succinic acid, which was exfoliated into ultrathin MOF nanosheets by immersed in a mixed solution of K_2_HPO_4_ and KH_2_PO_4_ (PBS, pH = 7.0). The inclusion of PBS solution particles (K^+^, H_2_PO_4_^−^, HPO_4_^2−^ and H_2_O) are inserted into the interlayer of the 2D MOFs to form hydrogen‐bonded inorganic layers, which not only provides proton source and conduction network, but also effectively regulates the energy states and band electron delocalization of the hybrid structure, realizing high‐efficiency mixed protonic–electronic electron conduction (**Figure** **1**).

**Figure 2:**
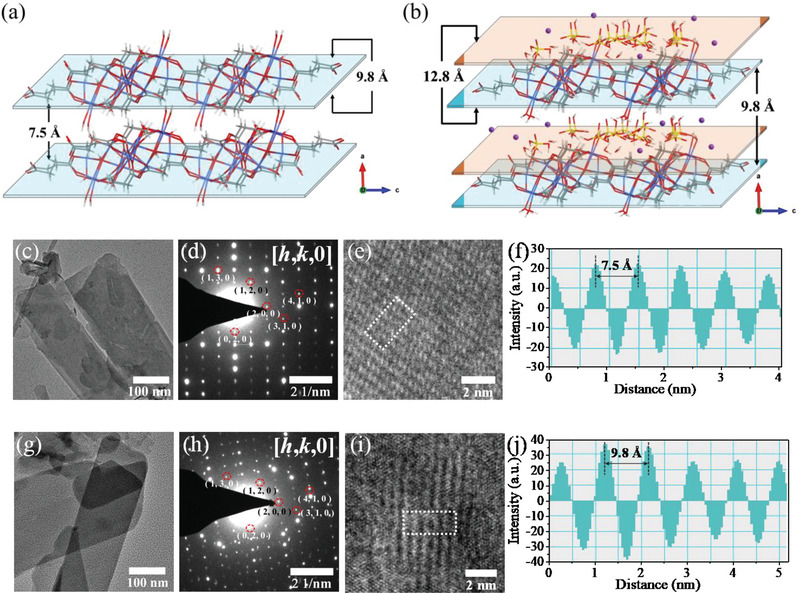
Key intralayer and interlayer parameters of a) **2D‐Co** and b) **2D‐Co‐NS‐PBS** (The K and the extra H ions are omitted). Low‐magnification TEM image of c) **2D‐Co‐NS** and g) **2D‐Co‐NS‐PBS**. SAED pattern of d) **2D‐Co‐NS** and h) **2D‐Co‐NS‐PBS**. HRTEM image of e) **2D‐Co‐NS** and i) **2D‐Co‐NS‐PBS**. f,j) Atom intensity profiles as outlined in e,j) dashed boxes, respectively.

The 2D MOFs crystal structure selected is [Co_7_(OH)_6_(H_2_O)_3_(C_4_H_4_O_4_)_4_]·7H_2_O^[^ ^19^ ^]^ (denoted as **2D‐Co**), which crystallizes in the monoclinic space group *P*21/*c* and its independent unit contains seven metal centers Co(II), four fully deprotonated ligands C_4_H_4_O_4_^2−^, six bridging OH^−^ and three coordinated H_2_O molecules, and some disordered guests H_2_O molecules (Figure S1, Supporting Information). In Figure S2 (Supporting Information), all the Co(II) show the coordination configuration of the octahedron, in which the adjacent hexamer unit [Co_6_(O)_14_(OH)_6_(H_2_O)_3_] is connected into (–Co–O–)_∞_ chair by the bridged of Co7. Then, the cobalt oxide chain (–Co–O–)_∞_ is connected by C_4_H_4_O_4_^2−^ to form 2D layer with a thickness of 9.8 Å corresponding to the [*h*,*k*,0] crystal face or *bc* plane, and the monolayer {Co_7_(OH)_6_(H_2_O)_3_(C_4_H_4_O_4_)_4_}_n_ is stacked along the direction of *d*_001_ to form **2D‐Co** with the interlamellar spacing of 7.5 Å (**Figure** **2**a; Figure S2, Supporting Information).

Generally, 2D bulk MOFs can be exfoliated into nanosheets while maintaining the precursor structure.^13^, ^20^ ^]^ Therefore, the microcrystals of **2D‐Co** with the morphology of nanometer ribbon (denoted as **2D‐Co‐Bulk**) can be also exfoliated into small nanosheets (denoted as **2D‐Co‐NS**), which maintains the structure of precursor of **2D‐Co** (Figure 2c; Figures S3–S8, Supporting Information). Transmission electron microscopy (TEM), energy‐dispersive spectrometry (EDS), and X‐ray photoelectron spectroscopy (XPS) indicated **2D‐Co‐NS** possesses Co, C, and O elements as same as the **2D‐Co‐Bulk**, which are distributed on the surface of the lamellar structure (Figures S9–S14, Supporting Information). Due to the single‐layered or few‐layered MOF nanosheets visibly and rapidly degraded during imaging by HRTEM,^[^ ^21^ ^]^ therefore the thicker nanosheets were selected for measurement to accurately determine the stacking mode of 2D materials.^[^ ^22^ ^]^ As shown in Figure 2d, the selected‐area electron diffraction (SAED) pattern of **2D‐Co‐NS** corresponds to the diffraction pattern of the [*h*,*k*,0] crystal face of the nanosheets, being in good agreement with the simulated SAED pattern of **2D‐Co** (Figure S15, Supporting Information). Furthermore, the distance between the adjacent layers of **2D‐Co‐NS** is ≈7.5 Å (Figure 2e,f), corresponding to the interlamellar spacing of crystallographic **2D‐Co** along the direction of *d*_001_. The tapping‐mode atomic force microscopy (AFM) image showed that the lamella of **2D‐Co‐NS** has large lateral dimension up to the micrometer size and the thickness is ≈1.0 nm (Figure S16, Supporting Information), equivalently to the thickness of 2D layer of **2D‐Co‐Bulk**. It indicates that **2D‐Co‐NS**, as the assembly unit of 2D layer of **2D‐Co**, is assembled into 3D structure by weak action along the direction perpendicular to the layer.

Noticeably, **2D‐Co‐Bulk** was immersed in 0.2 m PBS solution (pH = 7.0) for 12 h without ultrasound, which can transform to nanosheets similar to the morphology of **2D‐Co‐NS**, named as **2D‐Co‐NS‐PBS** (Figure 2g; Figures S17 and S18, Supporting Information). No cobalt element and succinic acid were detected in the solution of **2D‐Co‐Bulk** after immersed by inductively coupled plasma‐atomic emission spectrometry (ICP‐AES) and ^1^HNMR, respectively (Table S1 and Figure S19, Supporting Information). Contrastively, SEM and TEM revealed that the ultrasonic process cannot break the true morphologies of **2D‐Co‐NS‐PBS** (Figure S20, Supporting Information). By comparing the PXRD patterns of **2D‐Co‐Bulk** and **2D‐Co‐NS‐PBS** (Figure S3, Supporting Information), there is no significant difference in the peak patterns of their diffraction peaks, but the peaks of (002) and (012) decrease from 7.5^o^ and 8.5^o^ to 6.4^o^ and 7.4^o^, indicating that the latter has larger cells than that of the former, which implies the spatial position of the 2D MOF layer changes or new materials enters the 2D structure. TEM‐EDS and XPS indicated **2D‐Co‐NS‐PBS** has not only Co, C, and O elements of the precursor **2D‐Co‐Bulk**, but also K and P elements of the PBS solution, all the elements are distributed on the surface of the lamellar structure (Figures S21–S23, Supporting Information). Compared with **2D‐Co‐NS**, the SAED pattern of **2D‐Co‐NS‐PBS** appears a tremendous intensity modulation with alternating dark and bright diffraction spots at almost sites of [*h*,*k*,0] crystal face (Figure 2h). Meanwhile, the overall characteristic 2D metal–organic layer diffraction pattern remains unchanged, suggesting a stable host lattice and the absence of secondary phases. This suggests that the exotic species insert the interlamination of 2D MOFs, and the intercalation cannot change the stacking mode of metal–organic layers.^[^ ^23^ ^]^ Moreover, the distance between the adjacent layers of **2D‐Co‐NS‐PBS** (≈9.8 Å) is larger than that of **2D‐Co‐NS** (≈7.5 Å), and both of them are along the *d*_001_ of the referential **2D‐Co** crystal (Figure 2i,j), further confirming that the particles originated from PBS solution only merely insert into interlamination. Correspondingly, the layer thickness of **2D‐Co‐NS‐PBS** with ultrasonic dispersion is ≈1.3 nm (Figure S24, Supporting Information), being thicker than that of **2D‐Co‐NS** (≈1.0 nm) or the thickness of single layer of **2D‐Co**. Combining with infrared spectra (Figure S25, Supporting Information) and high‐resolution (HR) XPS (Figures S26 and S27, Supporting Information), the exotic species of **2D‐Co‐NS‐PBS** are further determined as H_2_PO_4_^−^ and HPO_4_^2−^ of PBS solution.^[^ ^24^ ^]^

**Figure 3:**
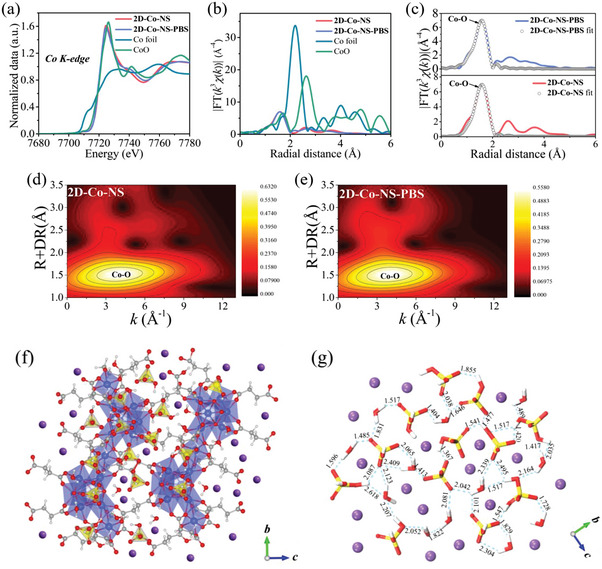
a) Normalized Co K‐edge XANES spectra and b) Fourier transform EXAFS spectra of Co‐based samples. The first shell fittings of the Fourier transform EXAFS spectra for c) **2D‐Co‐NS** and **2D‐Co‐NS‐PBS**. Results of wavelet Transform applied to EXAFS spectra of d) **2D‐Co‐NS** and e) **2D‐Co‐NS‐PBS**. f) The initial structure model of **2D‐Co‐NS‐PBS**. g) The initial structure model of hydrogen‐bonded inorganic layers. Dashed lines illustrate potential paths for proton transport. Distances are in Å. Cobalt, blue; carbon, dark gray; oxygen, red; phosphorus, yellow; hydrogen, light gray (partially omitted).

The refined structure of **2D‐Co‐NS‐PBS** was further determined by X‐ray absorption near‐edge structure (XANES), extended X‐ray absorption fine structure (EXAFS), and the corresponding R‐space analysis. As shown in **Figure** **3**a, the Co K‐edge XANES spectra of **2D‐Co‐NS‐PBS** exhibited characteristic edge absorptions at 7709.5 and 7724.8 eV, corresponding to the Co pre‐edge (Co1*s*–O2*p* hybrid orbitals) and Co1*s*–Co3*d* features, which is almost exactly coincident with the exfoliating precursor **2D‐Co‐NS** (7709.8 and 7724.6 eV). In addition, the valance state of Co are in the oxidation state of +2 before and after intercalation, which is consistent with the XPS result. The Fourier‐transformed (FT) EXAFS indicates that the coordination structure of Co(II) do not change (Figure 3b; Figure S28, Supporting Information). According to the fitting results, the local structure of Co(II) in **2D‐Co‐NS‐PBS** is almost indistinguishable from **2D‐Co‐NS** and consistent with the experimental data, while both the R‐space of them are 1.57 Å and attributed to the Co–O hexagon configurations (Figure 3c; Figures S29 and S30 and Table S2, Supporting Information). The wavelet transform analysis was performed on the experimental data,^[^ ^25^ ^]^ and no obvious difference in the **2D‐Co‐NS** and **2D‐Co‐NS‐PBS**, existing the Co–O signals similar to CoO, which can be verified by the FT EXAFS data and fitting structure (Figure 3d,e; Figures S31 and S32, Supporting Information). Moreover, both the electron paramagnetic resonance (EPR) spectrums of **2D‐Co‐NS** and **2D‐Co‐NS‐PBS** shows a strong signal at *g* = 1.9 (Figure S33, Supporting Information), suggesting the spin states of the metal's central ions have not changed. Due to the layer {Co_7_(OH)_6_(H_2_O)_3_(C_4_H_4_O_4_)_4_}_n_ of **2D‐Co‐NS‐PBS** does not change at all, the introduced ions K^+^, H_2_PO_4_^−^, and HPO_4_^2−^ are intercalated with the metal–organic layers.

The indexing and refinement of the PXRD patterns of **2D‐Co‐NS‐PBS** (without ultrasonic dispersion, the thicker of ≈10.8 nm, Figure S34, Supporting Information) were carried out using the Reflex module of Material Studio 5.0 (Figures S35 and S36, Supporting Information), which is well indexed in space group (*P*21/*c*) with cell parameter list as Table S3 in the Supporting Information. Based on all characterization results, we built an initial structure model for further calculation (Figure 3f). The refined structure of **2D‐Co‐NS‐PBS** implies the crystal symmetry of the intercalated layer (See in Figure 3g, the K^+^, H_2_PO_4_^−^, HPO_4_^2−^, and H_2_O form 2D layer by hydrogen bonding, denoted as **PBS‐NS**) is matched with that of {Co_7_(OH)_6_(H_2_O)_3_(C_4_H_4_O_4_)_4_}*_n_*, the metal–organic layers and intercalation can form a double‐layer heterojunction as the construct unit layer of 2D structure with the thickness and interlayer distance of 12.8 and 9.8 Å, respectively (Figure 2b). Because **PBS‐Ns**s additionally provides a proton source and hydrogen bond networks for proton transport in the host–guest architecture, which should be critical to driving proton conduction within solid‐state materials.

With reference to the ratio of MOF and inorganic salts in **2D‐Co‐NS‐PBS**, 115.9 mg **2D‐Co‐Bulk** was physically blending with 21.9 mg inorganic salts of K_2_HPO_4_ and KH_2_PO_4_ (*n*:*n* = 1:2). PXRD showed that the mixture contained **2D‐Co‐Bulk** diffraction peaks and inorganic salts diffraction peaks (23.9^o^, 45.6^o^, and 46.5^o^, corresponding to (200), (321), and (312) crystal planes of KH_2_PO_4_, respectively) (Figure S37, Supporting Information), which was different from that of **2D‐Co‐NS‐PBS** and no new phase created. The SEM images of the mixture showed that the morphology of the mixture was not nanosheets, but the nanoribbon similar to **2D‐Co‐Bulk**, and some random size ≈30 nm particles were adhered to its surface (Figure S38, Supporting Information). It suggested that physical mixing could not be similar to the immersion solution method for the formation of intercalated compounds. In addition, to study the effect of ions on the structure and morphology of **2D‐Co‐Bulk**, K_2_SO_4_, Na_2_SO_4_, and K_2_Cr_2_O_7_ were used to replace the mix inorganic salts of K_2_HPO_4_ and KH_2_PO_4_ without changing other conditions. The structure and morphology of **2D‐Co‐Bulk** did not change after immersing in the above comparative solution (Figures S39 and S40, Supporting Information). XPS and ICP‐AES analysis showed that no ions of immersed solution were detected in **2D‐Co‐Bulk** after immersing (Figures S41 and S42 and Table S1, Supporting Information), which further indicated that ions of immersed solution could not be inserted into the layers of MOFs, and the valence state of Co does not change.

**Figure 4:**
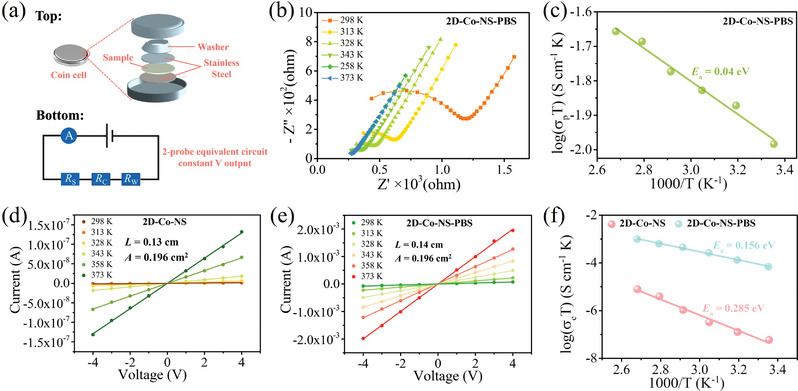
a) Top: schematic illustration of a home‐made device for manufacturing and measuring a two‐contact probe devices; Bottom: equivalent circuit for a two‐contact probe device (*R*_S_ = resistance of sample of interest, *R*_C_ = contact resistance; *R*_W_ = wire resistance). b) Nyquist plots with different temperature for **2D‐Co‐NS‐PBS** under 99% RH. c) Arrhenius plots of proton conductivity for **2D‐Co‐NS‐PBS** under 99% RH. Representative *I*–*V* curves with different temperature for d) **2D‐Co‐NS** and e) **2D‐Co‐NS‐PBS** under 99% RH, the solid lines correspond to linear fits to the data. f) Arrhenius plots of electron conductivity for **2D‐Co‐NS** and **2D‐Co‐NS‐PBS** under 99% RH.

In order to simulate practical application, we prepared the ion intercalation MOFs of coin cell as the ion separator device (**Figure** **4**a), and then carried out electrochemical impedance spectroscopy (EIS) measurement to investigate the ability of **PBS‐NS** intercalated metal–organic film as a solid electrolyte. The EIS pattern of **2D‐Co‐NS** is no semicircle observed (Figure S43, Supporting Information), indicating the negligible proton conductivity of the parent frameworks, which is due to the absence of a proton conduction source in the structure. Correspondingly, **2D‐Co‐NS‐PBS** containing **PBS‐NS** as a proton conduction source shows typical proton conductivity and temperature‐dependent proton conductivity, which was determined by the semicircle in Nyquist plot and the relevant equivalent circuit model (Figure 4b). The proton conductivity of **2D‐Co‐NS‐PBS** was measured to be 1.70 × 10^−6^ S cm^−1^ at 25 °C and 99% relative humidity (RH), which increases as the temperature increases, obtaining the optimal conductivity of 2.02 × 10^−5^ S cm^−1^ at 100 °C and 99% RH. The activation energies (*E*_a_) of the materials derived from Arrhenius plots is found to be 0.04 eV for **2D‐Co‐NS‐PBS** (Figure 4c), which lies within the range corresponding to the conventional Grotthuss mechanism of MOFs (*E*_a_ ≤ 0.4 eV).^[^ ^10b^ ^]^ Based on the proton conduction mechanism of **2D‐Co‐NS‐PBS**, the protons originate from **PBS‐NS** and migrate directionally along the 2D hydrogen bond networks constructed by metal–organic layer and inorganic layer, which verifies the rationality of 2D heterojunction structure.

Subsequently, the electrochemical workstation was replaced with Keithley 6517B instrument to test the electronic conductivity of self‐made Coin Cell at 99% RH. It is a simple double‐contact probe device, similar to Diaca's home‐built apparatus for fabricating and measuring two‐contact probe pressed pellet devices in situ.^[^ ^26^ ^]^ In Figure 4d,e, both the electron conductivity of **2D‐Co‐NS** and **2D‐Co‐NS‐PBS** increases with the rising of temperature in the range of 25–100 °C, corresponding to the electron conductivity increased from 2.09 × 10^−10^ to 2.12 × 10^−8^ S cm^−1^ and 2.03 × 10^−5^ to 3.84 × 10^−4^ S cm^−1^, respectively. The Arrhenius plots of **2D‐Co‐NS** and **2D‐Co‐NS‐PBS** belongs to linear relationships and the *E*_a_ is 285 eV and 156 eV (Figure 4f), which indicates that the semiconductor‐like behavior of these materials.^[^ ^27^ ^]^ The 2D intercalated materials originating from the insertion of inorganic layer **PBS‐NS** between the metal‐organic layer {Co_7_(OH)_6_(H_2_O)_3_(C_4_H_4_O_4_)_4_}*_n_* effectively tuned the structure's electron conductivity, increasing the conductivity by 4–5 orders of magnitude. The MOFs with inorganic ions intercalation exhibits excellent proton–electron dual conduction, which is comparable to most of the other material that has been reported (see in the Table S4 in the Supporting Information). In addition, the energy bandgap (*E*_g_) of **2D‐Co‐NS** (2.16 eV) and **2D‐Co‐NS‐PBS** (1.80 eV) can be derived from their UV–vis–NIR spectra (Figure S44, Supporting Information), which is consistent with the order of their electronic conductivity, further indicates that the extraneous intercalation has a manipulable regulatory effect on the electronic structure of the metal–organic layer of the parent. Further, **2D‐Co‐NS** displays high stability electrochemical testing, as demonstrated by the negligible change of PXRD pattern, XPS profile, and EPR analysis (Figures S45–S48, Supporting Information). Noticeably, the PXRD peaks (002) and (012) of **2D‐Co‐NS‐PBS** are corresponding to a metal–organic layer perpendicular to the intercalated structure, they are broadened and decreased after the electrochemical testing (Figure S45, Supporting Information), implying that the proton conduction intensifies the disordered accumulation of 2D metal–organic layers. Since the PXRD of **2D‐Co‐NS‐PBS** was almost unchanged after 72 h fumigation at 100 °C and 99% RH (Figure S45, Supporting Information), which suggests electrochemical testing is the main factor leading to the crystal degradation. By successive electrochemical test self‐made Coin Cells of **2D‐Co‐NS‐PBS** at 99% RH, both the proton conductivity and the electron conductivity were found to decrease gradually (Figures S49 and S50, Supporting Information). The values of proton conductivity decreases from 1.70 × 10^−6^–2.02 × 10^−5^ S cm^−1^ to 4.25 × 10^−7^–5.85×10^−6^ S cm^−1^ (the range of 25–100 °C) after twice electrochemical testing; corresponding to the electron conductivity decreases from 2.03 × 10^−5^–3.84×10^−4^ to 7.12 × 10^−6^–4.85 × 10^−5^ S cm^−1^. Due to the disorder of layer‐layer stacking leads to the hydrogen bond chain be destroyed and the proton conductivity decreases, and the non‐crystallizing also leads to the decrease of its electron conductivity. Meanwhile, the XPS profile and EPR analysis reveal that the valence states and spin states of metal center in **2D‐Co‐NS‐PBS** does not change before and after the electrochemical test (Figures S46–S48, Supporting Information), which further indicates that the noncrystallizing resulted in the decrease of electrical conductivity.

**Figure 5:**
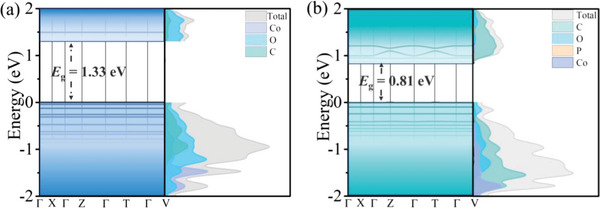
Calculated energy bands and projected density of states (DOS) of a) **2D‐Co‐NS**, and b) **2D‐Co‐NS‐PBS**. Gray curves represent total DOS. Blue, teal, yellow, red, and black curves represent projected DOS of Co, C, P, and O, respectively.

The mechanism of electron band conduction in the metal‐organic layer before and after PBS intercalation was further investigated by density functional calculation. It reveals the intrinsic electronic band structure of **2D‐Co‐NS** and **2D‐Co‐NS‐PBS** (**Figure** **5**), the former is relatively wide with the calculation value *E*_g_ of 1.33 eV, higher than that of the latter (0.81 eV), which is consistent with the optical band gap obtained by electronic spectroscopy. Furthermore, the density of states (DOS) indicates that the valence bond (VB) is mainly composed of Co and O centered orbitals (Figure 5), confirming that the (–Co–O–)_∞_ chain in the two structures is the main charge transport pathway. Since the low conduction bands of these two structures are dominated by ligand orbitals, the work function (*Φ*, the absolute energy scale are aligned to vacuum according to Aron Walsh, et al.^[^ ^28^ ^]^) decreases from 5.14 to 4.78 eV (Figure 5; Figure S51, Supporting Information) due to the enhanced orbital contribution of the metal–organic layer ligand by **PBS‐NS** intercalation, which is consistent with the *E*_a_ obtained from the variable‐temperature electronic conductivity measurement. It indicates that intercalation effectively regulates the surface electronic structure of the metal–organic layer and indirectly regulates the intrinsic electron conduction ability of the structure.

## 3 Conclusion

In summary, we developed a new strategy that the guest particles of inorganic ions and/or molecules insert into the interlamination of neutral host framework 2D MOFs, manufacturing the host–guest architecture of 2D metal–organic layers and hydrogen‐bonded inorganic layers. In this architecture, the hydrogen‐bonded inorganic layers offers the proton source and proton transfer path, while the 2D metal–organic layers provides the electron conduction path to ensure the proton and electron conductivity of the 2D hybrid networks, respectively. Moreover, the electronic structure of host frameworks of MOFs can be accurately regulated by the intercalation, to increase the electronic conductivity of intrinsic MOFs. This work expands the category of MPECs materials, as well as the fundamental understanding of structure–property relationship involved mixed conduction has further investigated.

## Conflict of Interest

The authors declare no conflict of interest.

## Supporting information

Supporting Information

## Data Availability

The data that support the findings of this study are available in the supplementary material of this article.
