# Supplementary material for: A Mixed Protonic–Electronic Conductor Base on the Host–Guest Architecture of 2D Metal–Organic Layers and Inorganic Layers
Source: Adv Sci (Weinh). 2023 Apr 19;10(17):2205944. doi: 10.1002/advs.202205944 (PMC10265077; doi:10.1002/advs.202205944)
Supplement: Supplementary file 1 — Supporting Information [file ADVS-10-2205944-s001.pdf]

ADVANCED SCIENCE
Open Access

Supporting Information

for *Adv. Sci.*, DOI 10.1002/advs.202205944

A Mixed Protonic–Electronic Conductor Base on the Host–Guest Architecture of 2D Metal–Organic Layers and Inorganic Layers

Xing-Lu He, Bing Shao, Rui-Kang Huang, Min Dong, Yu-Qing Tong, Yan Luo, Ting Meng, Fu-Jie Yang, Zhong Zhang and Jin Huang**

Supporting Information for

A Mixed Protonic−Electronic Conductor Base on the Host-Guest Architecture of Two−Dimensional Metal−Organic Layers and Inorganic Layers

Xing-Lu He‡,[a] Bing Shao‡,[a,b] Rui-Kang Huang‡,[c] Min Dong,[a] Yu-Qing Tong,[b] Yan Luo,[a] Ting Meng,[a] Fu-Jie Yang,[d] Zhong Zhang,*[b] Jin Huang*[a]

[a]*Guangxi Key Laboratory of Bioactive Molecules Research and Evaluation, College of Pharmacy, Guangxi Medical University, Nanning, 530021, P. R. China*

[b]*School of Chemistry and Pharmaceutical Sciences, Guangxi Normal University, Guilin, 541004, P. R. China*

[c]*Research Institute for Electronic Science, Hokkaido University, Sapporo, 001-0021, Japan*

[d]*College Chemistry and Chemical Engineering, Zhongkai University of Agriculture and Engineering, Guangzhou 510275, P. R. China.*

*E-mail: huangjin@mailbox.gxnu.edu.cn; zhangzhong@mailbox.gxnu.edu.cn.

Experiment Section

Experimental materials and instruments: All metallic salts and reagents are purchased from the market and are used directly without further treatment. Powder X-ray diffraction (PXRD) patterns in the range of 3 to 60° at a rate of 3° min^{-1} were collected using a Rigaku D/max diffractometer apparatus (Cu Kα, λ = 1.54056 Å). Scanning electron microscope (SEM) images were acquired with an FEI-Quanta 200 equipment working at 20/30 kV acceleration voltage. The transmission electron microscope (TEM) images were recorded by FEI-Talos-200S dry aberration correction instrument. The Atomic Force Microscope (AFM) images were obtained by Bruker apparatus. X-ray photoelectron spectroscopy (XPS) was obtained by the Thermo Scientific Escalab 250xi apparatus. Fourier infrared spectroscopy (FT-IR) in the 400-4000 cm^{-1} region were collected by the instrument of Nicolet 360 FT IR spectrometer. Raman spectra in the wavenumber range of 400-4000 cm^{-1} were acquired with the Renishaw inVia. The inductively coupled plasma atomic emission spectrometry (ICP-AES) detecting metal element analysis were obtained by the PekinElmer Optima 8300 apparatus. ^{1}H NMR spectra were recorded at ambient temperature using 400 MHz spectrometers. Solid-state UV data were acquired on a Shimadzu UV-2600 UV-Vis spectrometer. The electronic conductivity data was obtained by Keithley 6517B instrument. The proton conductivity data was acquired with the electrochemical workstation (CHI760E). Co K-edge Fourier transform extended X-ray absorption fine structure (EXAFS) carried out at the XAS station (BL14W1) of the Shanghai Synchrotron Radiation Facility (SSRF).

Synthesis of 2D-Co-Bulk. The succinic acid (0.98 g, 8.31 mmol) was dissolved in 33.3 mL H_2O, then $Co(OH)_2$ (1.55 g, 16.67 mmol) was added and transferred to a 48 mL Schlenk flask, which was sealed under N_2 for 5 minutes and heated at 150 °C stirring for 24 hours. The resulting precipitate was filtered, then washed three times with H_2O and dried in the air (yield *ca.* 95%).

Preparation of 2D-Co-NS. The **2D-Co-Bulk** powder was ultrasonically dispersed in mixture of H_2O/MeOH (*v*/*v* = 1:1) for 60 min, washed with distilled

water and methanol for five times, and finally dried at room temperature.

Preparation of 2D-Co-NS-PBS. The **2D-Co-Bulk** powder was immersed in 0.2 M PBS solution (pH = 7.0). The pink powder turned to purple after 30 minutes. The precipitation was filtered after 12 hours, washed with distilled water for five times, and finally dried at room temperature.

Scanning electron microscopy (SEM) measurement. **2D-Co-NS** and **2D-Co-NS-PBS** were dispersed in methanol and aqueous solution (*v*/*v* = 1:1) by ultrasound for 30 minutes, respectively, and then stood for 5 min. Suck the supernatant and dropped it on the SiO_2/Si slices by capillary, dried at room temperature and characterized by SEM. Similarly, **2D-Co-Bulk** and **2D-Co-Bulk** were soaked in K_2SO_4, Na_2SO_4 and $K_2Cr_2O_7$ solutions obtained samples that were similar to the preparation method of **2D-Co-NS** except that ultrasound was not required.

Transmission electron microscopy (TEM) measurement. **2D-Co-NS** and **2D-Co-NS-PBS** were ultrasonically dispersed in mixture of H_2O/MeOH (*v*/*v* = 1:1) for 30 min, respectively, then let it stand for 5 minutes. suck the supernatant with a pipette (10 μL) and dropped the double copper mesh carbon film, which was dried at room temperature and characterized by TEM. Similarly, preparation method of **2D-Co-Bulk** was a similar way to that described for **2D-Co-NS** except that ultrasound was not required.

Atomic Force Microscope (AFM) measurement. **2D-Co-Bulk**, **2D-Co-NS** and **2D-Co-NS-PBS** were dispersed by ultrasound in mixture of H_2O/MeOH (*v*/*v* = 1:1) for 30 min, respectively. The supernatant droplets were absorbed with a pipetting gun (10 mL) for spin coating on a SiO_2/Si slice. AFM characterization was performed after drying at room temperature.

Calibration of selected area electron diffraction (SAED). The diffracted electron beam from the sample comes from a two-dimensional network of atoms. It has translational symmetry in two directions, denoted by unit vectors a and b, respectively. These two vectors define the plane of the two-dimensional network, the two-dimensional plane has the thickness of a single layer, and the diffracted electron beam is also irradiated vertically on this plane. According to the crystal plane required

for structural analysis, the crystal data (CCDC: 268305) of the sample was imported into the Single Crystal software, exported the electron diffraction points of the desired crystal plane with Crystal Maker, and then use Photoshop software to mark the crystal plane.

The inductively coupled plasma atomic emission spectrometry (ICP-AES) measurement. Considering the metal element content of **2D-Co-NS-PBS**, **2D-Co-Bulk** (10 mg) and **2D-Co-NS-PBS** (10 mg) were dissolved in 5% HNO_3 (5 mL) matrix and H_2O_2 (10 mL) respectively, then diluted to 1000 mL and analyzed with a Co internal standard against a 6-point standard curve over the range from 0.1 ppb to 500 ppb. The correlation coefficients of all correlation analyses were >0.9997, and each sample was tested three times in parallel, and each sample was repeated several times. Similarly, the ICP-AES tests for samples obtained by immersing **2D-Co-Bulk** in solutions K_2SO_4, Na_2SO_4 and $K_2Cr_2O_7$ were performed in the same way as **2D-Co-Bulk** and **2D-Co-NS-PBS** methods.

EXAFS curve fitting details. Data reduction, data analysis, and EXAFS fitting were performed and analyzed with the Athena and Artemis programs of the Demeter data analysis packages[*J. Synchrotron Rad.* **2005**, *12*, 537] that utilizes the FEFF6 program[*Phys. Rev. B* **1995**, 52, 2995] to fit the EXAFS data. The energy calibration of the sample was conducted through a standard Co foil, which as a reference was simultaneously measured. A linear function was subtracted from the pre-edge region, then the edge jump was normalized using Athena software. The $\chi(k)$ data were isolated by subtracting a smooth, three-stage polynomial approximating the absorption background of an isolated atom. The $k3$-weighted $\chi(k)$ data were Fourier transformed after applying a Hanning window function (Δk = 1.0). For EXAFS modeling, The global amplitude EXAFS (CN, R, σ^2 and ΔE_0) were obtained by nonlinear fitting, with least-squares refinement, of the EXAFS equation to the Fourier-transformed data in R-space, using Artemis software, EXAFS of the Co foil is fitted and the obtained amplitude reduction factor S_0^2 value (0.792) was set in the EXAFS analysis to determine the coordination numbers (*CNs*) in the Co-O scattering path in sample. For Wavelet Transform analysis, the $\chi(k)$ exported from Athena was imported into the

Hama Fortran code.[*Phys. Rev. B* **2005**, 71, 094110] The parameters were listed as follow: *R* range, 1-3.5 Å, *k* range, 0-13.0 $Å^{-1}$ for sample (0-13.0 $Å^{-1}$ for Co foil and CoO); *k* weight, 2; and Morlet function with κ=10, σ=1 was used as the mother wavelet to provide the overall distribution.

X-ray structure analysis. PXRD data of **2D-Co-NS-PBS** for Pawley and Rietveld refinements were collected on a Rigaku D/max X-ray powder diffractometer (Cu Kα, λ = 1.54056 Å) with a scanning speed of 1 ^{o}/min. All the indexing and refinement were performed by the Reflex plus module of Material Studio 5.0. The initial structural model of **2D-Co-NS-PBS** for Rietveld refinements was obtained from the crystal structure of **2D-Co (**CCDC: 268305). The pseudo-Voigt profile parameters, background parameters, the cell parameters, the zero point of the diffraction pattern, the global isotropic atom displacement parameters, the Berar-Baldinozzi asymmetry correction parameters, and the March-Dollase preferred orientation correction parameters were optimized step by step to improve the agreement between the calculated and the experimental powder diffraction patterns.

Electrochemical testing. An appropriate amount of the sample was ground uniformly and finely in a mortar, then put into a mold with a diameter of 1.58 cm, then pressed into a dense wafer with a thickness of 0.026 cm and an area of 1.96 cm^2 under 200 Mpa. We used the **s**elf-made coin cell for electrochemical testing. The **s**elf-made coin cell consists of the washer and two polymer-supported stainless steel coils. The diameter of the washer is the same as the inner diameter of the stainless steel coils (1.58 cm in diameter). In addition, the shell of self-made coin cell has holes drilled into it to allow humidity control. We put the dense wafer into the **s**elf-made coin cell and placed in an oven with adjustable humidity and temperature. Both ends of the Coin Cell respectively were respectively connected with silver glue and gold wire to concatenate the electrochemical apparatus for proton conductivity testing. Subsequently, the electrochemical workstation was replaced with Keithley 6517B instrument to test the electronic conductivity of self-made Coin Cell at 99% RH.

Electrochemical impedance spectroscopy (EIS) under different conditions (temperature range 298K~373k, relative humidity 99%). The charge conducted by

proton was a sinusoidal AC signal input (frequency 10^{-1}-10^{6} Hz and amplitude 50 mV) in the electrochemical workstation (CHI760E) when the DC static voltage is 0, then using the ZView2 program to output the arc region and the linear state composition, namely the Nquist plot. Finally, the radius of the arc part, that was, the *R* value of the ion transfer resistance, was obtained by the equivalent circuit fitting. The proton conductivity of the sample is calculated as follows[*Chem. Rev.* **2020**, *120*, 8416]:

$$\sigma_{\mathrm{i}} = \frac{L}{RA} \tag{1}$$

where σ_{i} is the proton conductivity, *R* is ion transfer resistance, *L* is the length and *A* is cross-sectional area through the sample to be tested.

The activation energy (E_{a}) of the sample was calculated through the Arrhenius equation:

$$\sigma_{\mathrm{i}} = \frac{B}{T}\exp\left(-\frac{E_a}{kT}\right) \text{ or } \ln(\sigma_{\mathrm{i}}T) = -\frac{E_a}{kT} + \ln B \tag{2}$$

where *B* is pre-exponential factor, E_{a} is the Arrhenius activation energy, *k* is Boltzmann constant, and *T* is the thermodynamic temperature.

Electrical conductivity, σ_{e}, measures the ability of a material to conduct electrical current. Measuring σ_{e} usually involves connecting the material under test to an electronic apparatus, typically a resistor. and measuring the electrical conductance (*G*), length (*L*), and cross-sectional area (*S*) of the channel. The electrical conductance is generally obtained by fitting the linear region of the current voltage (*I*-*V*) curve using Ohm's law[*J. Am. Chem. Soc.* **2016**, *138*, 14772].

$$\sigma_e = G\frac{L}{S} = \frac{I}{V} \times \frac{L}{S} \tag{3}$$

Calculation of band gap. Due to the different conductivity of metal conductors, semiconductors and insulators, their energy band structures are also different. The energy bands of metals are overlapped or half-filled with state, so there are both electrons and empty energy state in one energy band. The energy band where free electrons exist is called the conduction band, free holes exist is named the valence band, and electrons can move freely under the action of an electric field, which results in high conductivity. The conductivity of insulators and semiconductors is essentially

the same, the only difference is the difference in the band gap. To become free electrons or holes, the bound electrons must obtain enough energy to transition from the valence band to the conduction band, and the minimum value of this energy is the band gap. The band gap is an important parameter to measure the conductivity of semiconductor materials. According to the formula proposed by Tauc, Davis and Mott *et al*. Tauc plot.

$$(\alpha hv)^{1/n} = W(hv\text{-}E_g)$$

Among them, α is the absorption index, h is Planck's constant, v is the frequency, W is the absorbance, E_g is the forbidden band width of the semiconductor, n is related to the type of semiconductor, n = 1/2 for direct bandgap semiconductors; indirect bandgap semiconductors n = 2. Take hv as the abscissa and $(\alpha hv)^{1/n}$ as the ordinate to draw a graph, make a tangent, and the value that intersects with the abscissa is E_g.

Calculation of electronic band structure and density of states. First-principles calculations for structural optimization and analysis of the electronic properties were performed with the Vienna ab initio simulation package using the projector-augmented wave method.[*Phys. Rev. B* **1996**, *54*, 11169] The cutoff points for the kinetic energy and k-point spacing were set to 500 eV and approximately 0.1 $Å^{-1}$, respectively. During the optimization of the crystal structures, Gaussian smearing with a width of 0.2 eV was adopted for the integral over the Brillouin zone. For the exchangecorrelation energy within the density functional theory, the PBEsol functional, [*Phys. Rev. Lett.* **2008**, *100*, 136406] which revises the Perdew Burke-Ernzerhof–generalized gradient approximation functional to improve the equilibrium properties of solids and surfaces, was applied. For the electronic structure calculations, the tetrahedron method with Blöchl corrections and the HSE06 functional were used for the integral over the Brillouin zone and the exchange-correlation energy, respectively.[*J. Chem. Phys.* **2003**, 118, 8207; *J. Chem. Phys.* **2006**, *124*, 219906]

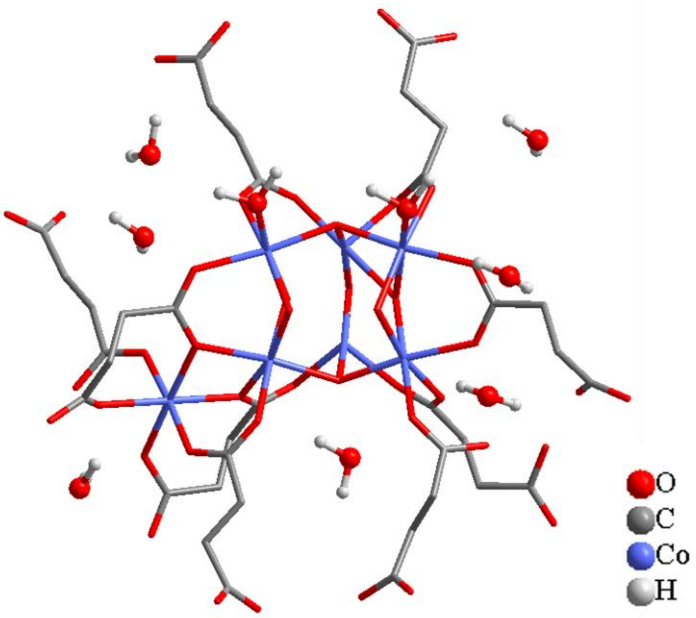

Figure S1. The asymmetric unit of **2D-Co**.

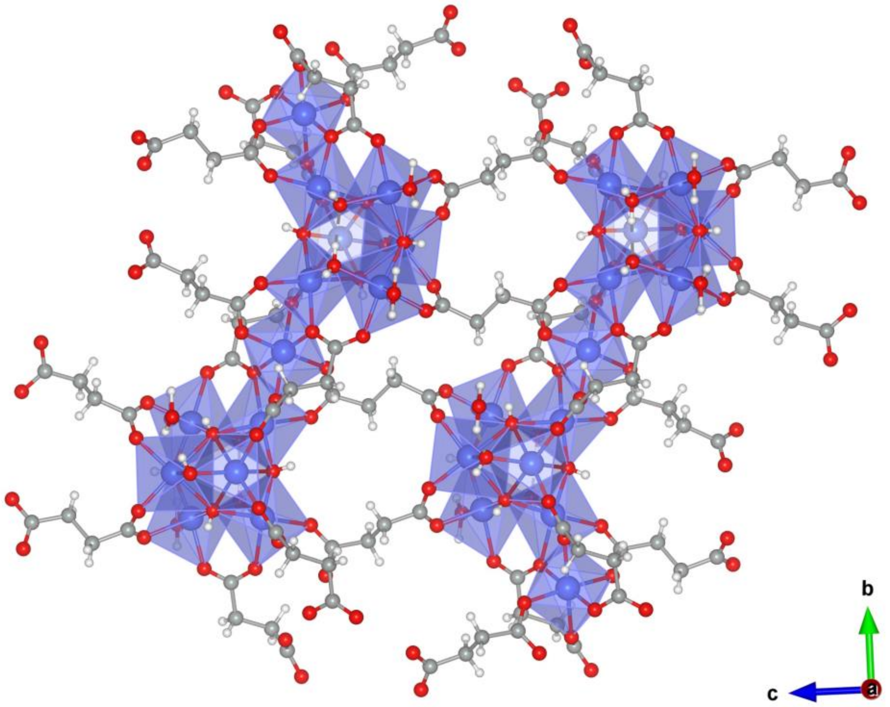

Figure S2. $\{Co_7(OH)_6(H_2O)_3(C_4H_4O_4)_4\}_n$ of **2D-Co** layer viewing perpendicular to the *bc*-plane.

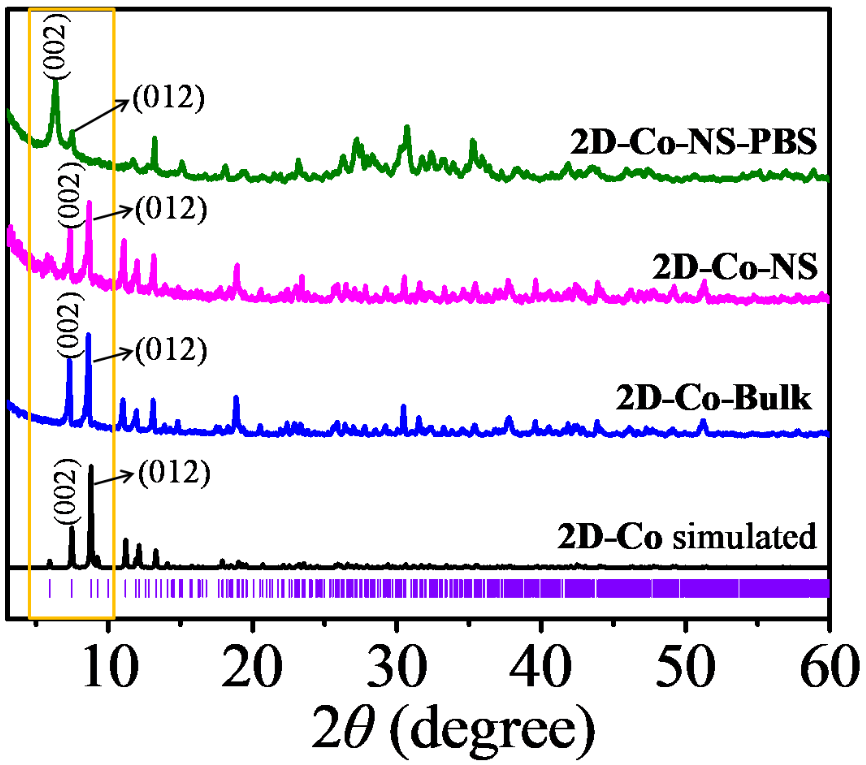

Figure S3. PXRD patterns of **2D-Co**, **2D-Co-Bulk**, **2D-Co-NS** and **2D-Co-NS-PBS**.

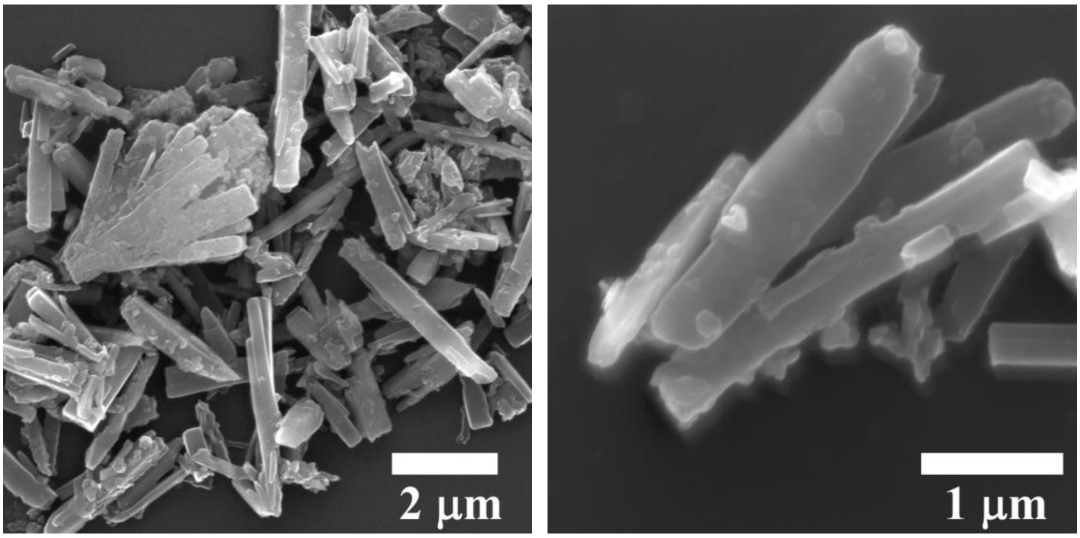

Figure S4. SEM images of **2D-Co-Bulk**.

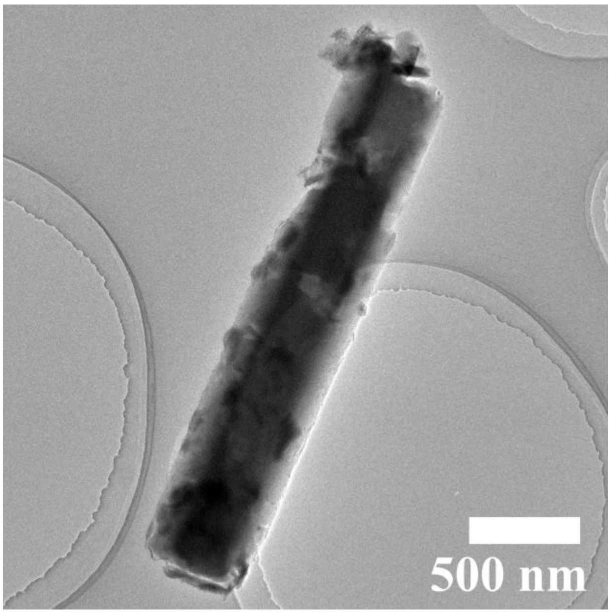

Figure S5. TEM image of **2D-Co-Bulk**.

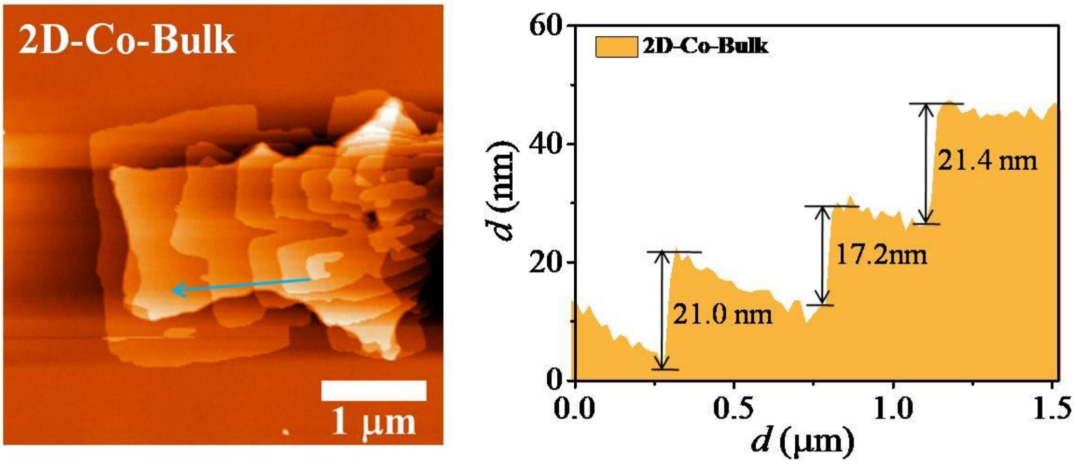

Figure S6. AFM images and height profiles of **2D-Co-Bulk**.

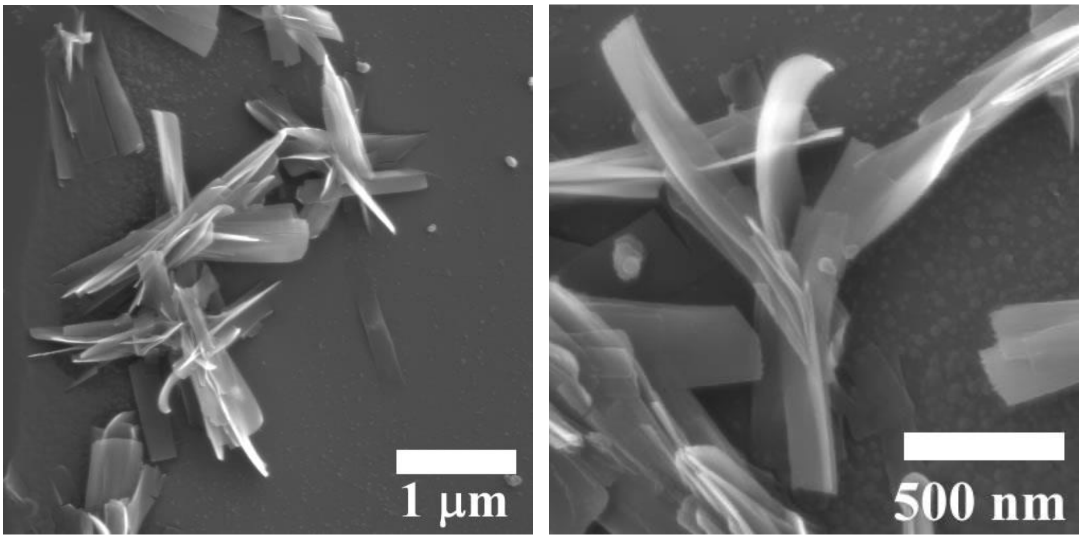

Figure S7. SEM images of **2D-Co-NS**.

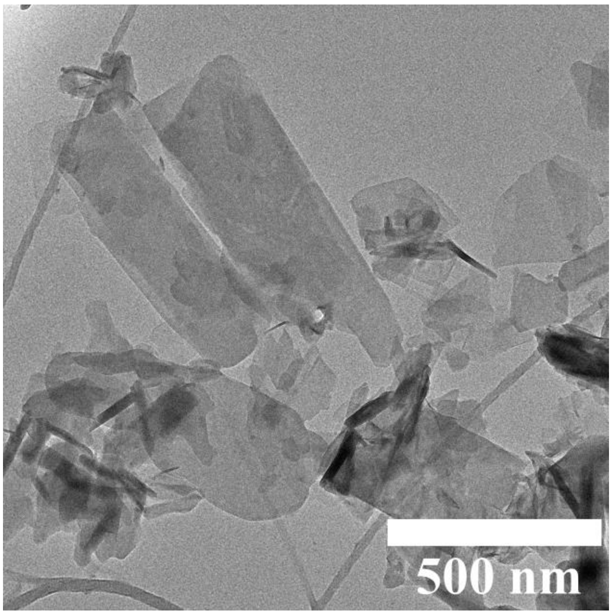

Figure S8. TEM image of **2D-Co-NS**.

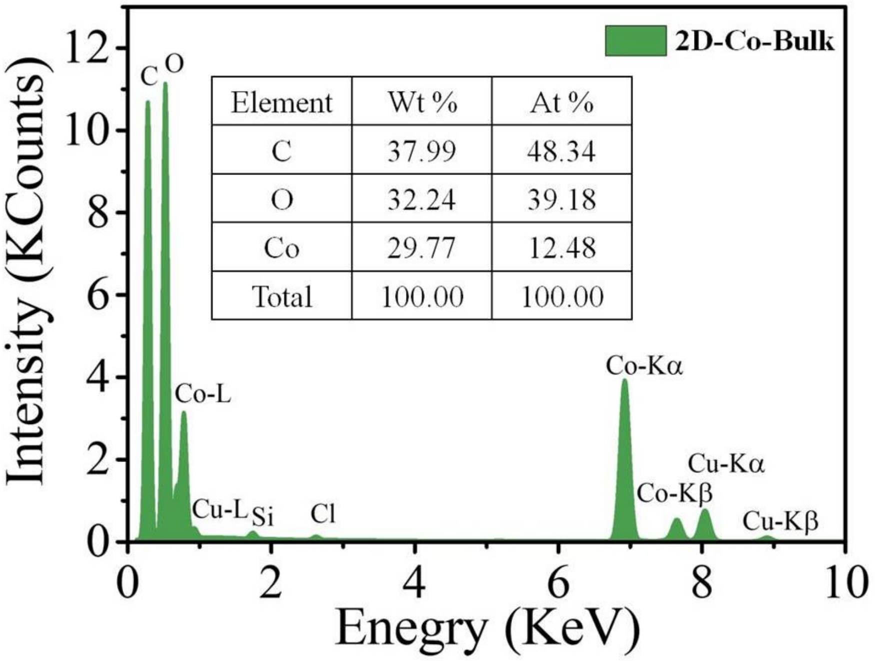

Figure S9. EDS spectrum of **2D-Co-Bulk**.

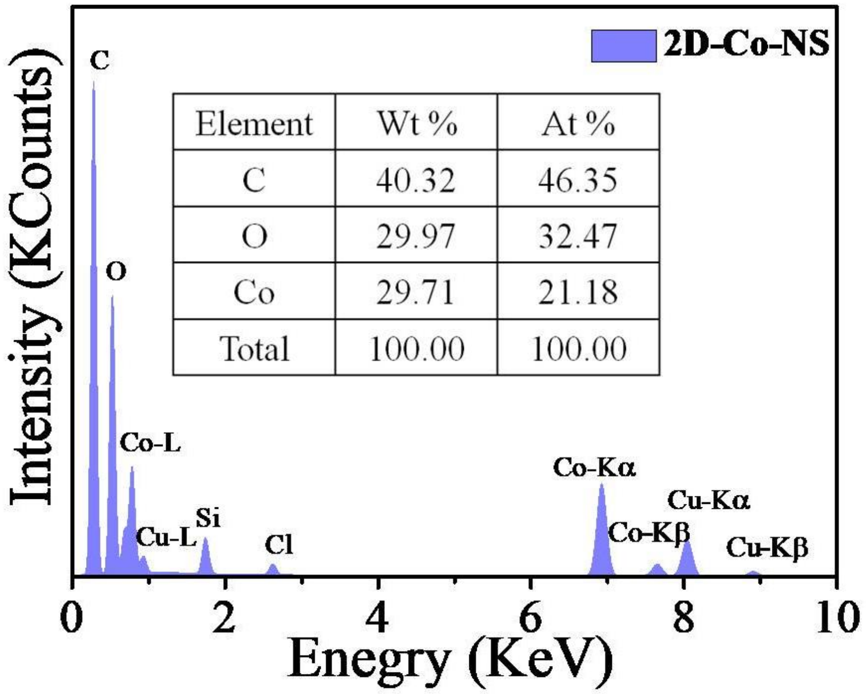

Figure S10. EDS spectrum of **2D-Co-NS**.

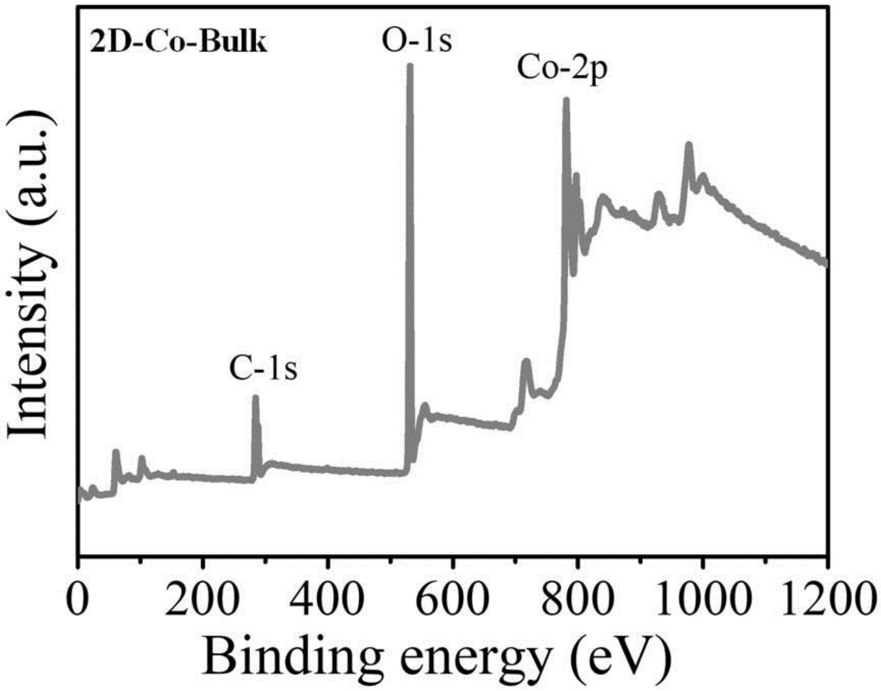

Figure S11. XPS spectrum of **2D-Co-Bulk**.

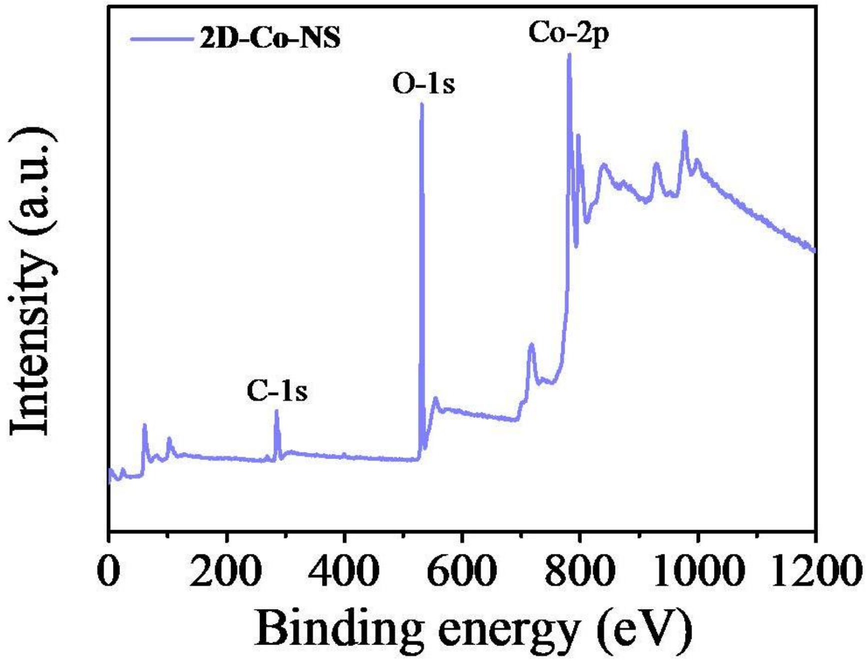

Figure S12. XPS spectrum of **2D-Co-NS**.

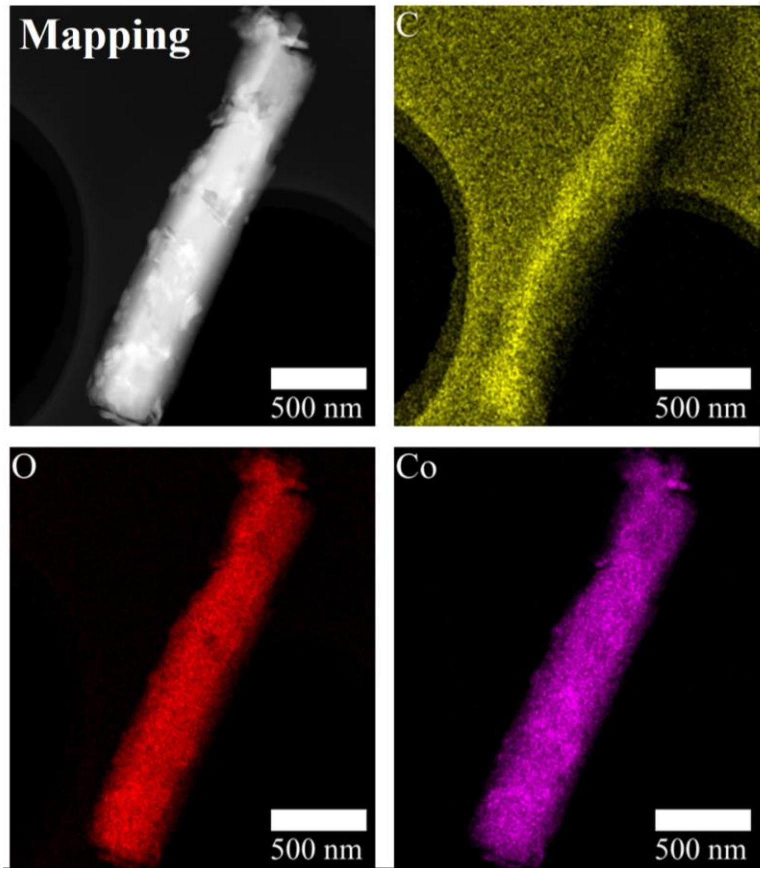

Figure S13. TEM-EDS mapping images of **2D-Co-Bulk**.

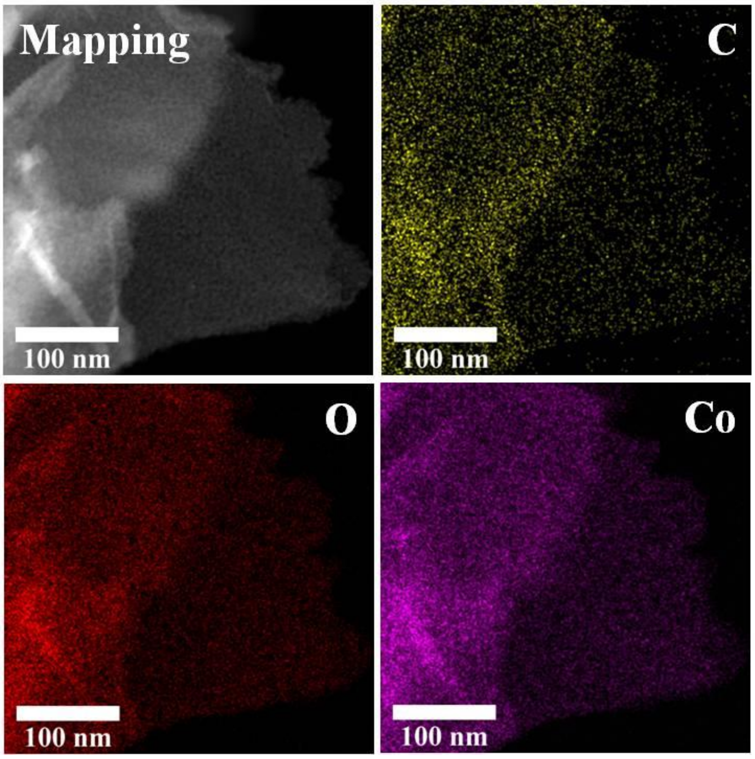

Figure S14. TEM-EDS mapping images of **2D-Co-NS**.

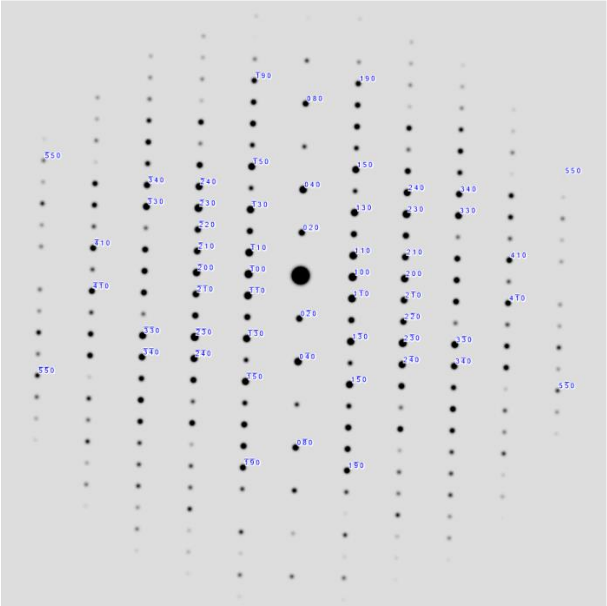

Figure S15. X-ray diffraction points in the [*h*,*k*,0] of **2D-Co**.

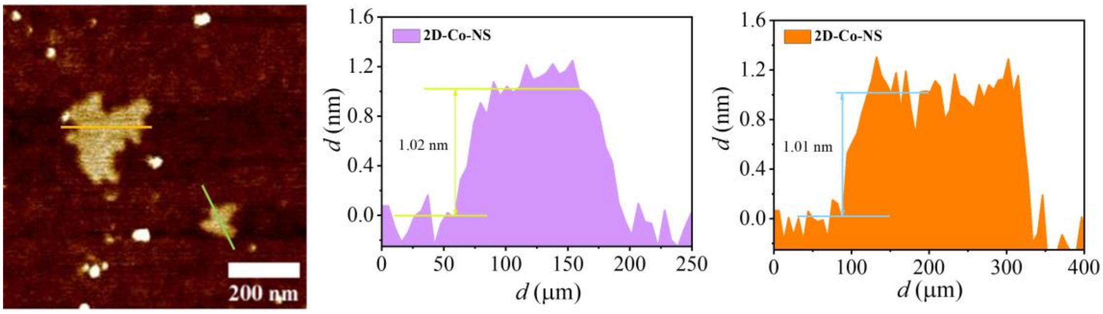

Figure S16. AFM images and height profiles of **2D-Co-NS**.

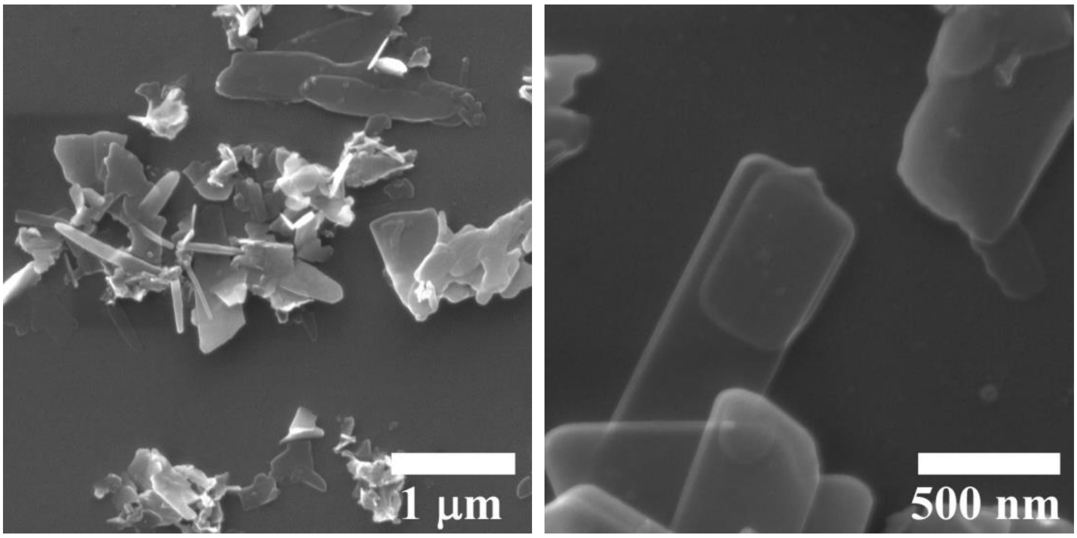

Figure S17. SEM images of **2D-Co-NS-PBS**.

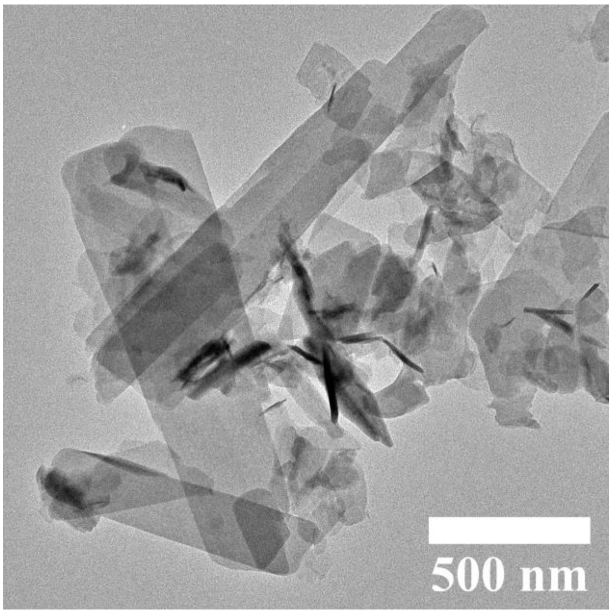

Figure S18. TEM image of **2D-Co-NS-PBS**.

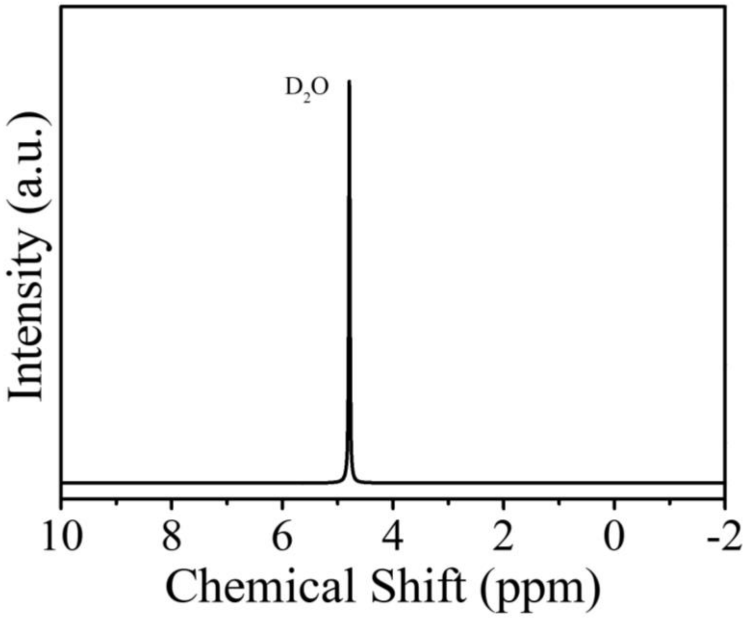

Figure S19. ^{1}HNMR of the solution of **2D-Co-Bulk** immersed for 6 hours.

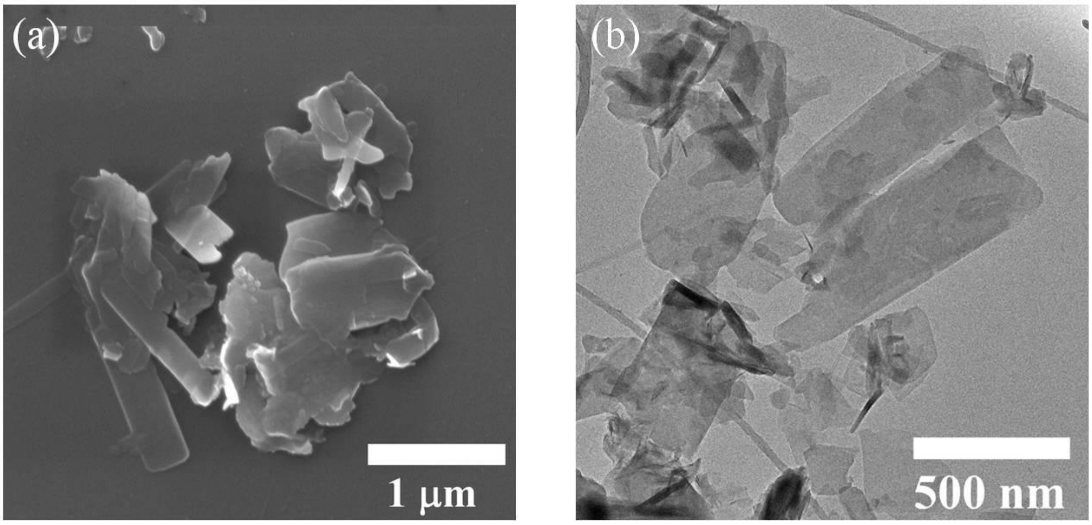

Figure S20. (a) The SEM and (b) TEM images of **2D-Co-NS-PBS** after ultrasonic process.

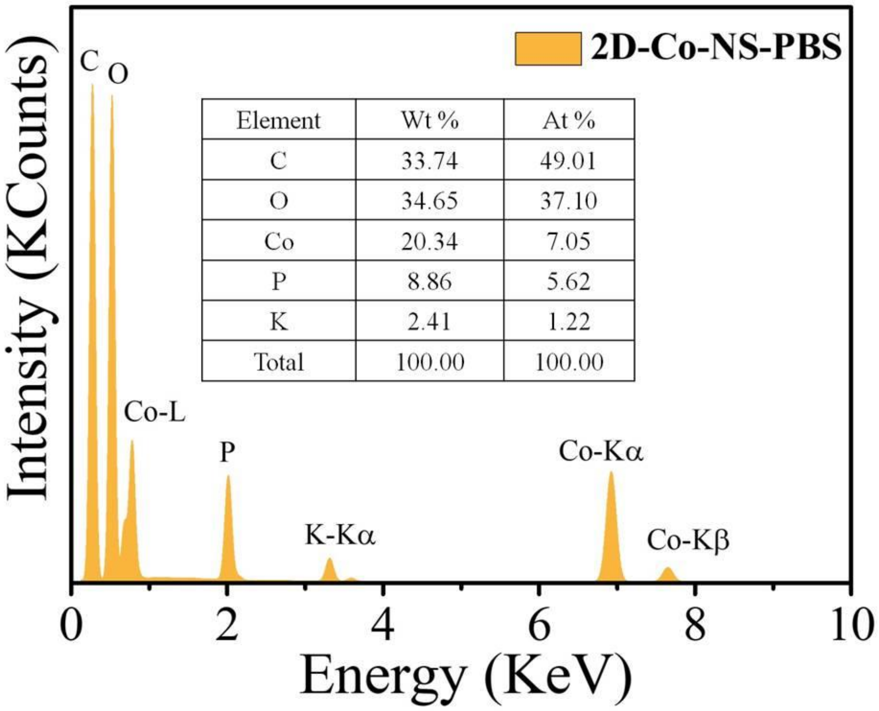

Figure S21. EDS spectrum of **2D-Co-NS-PBS**.

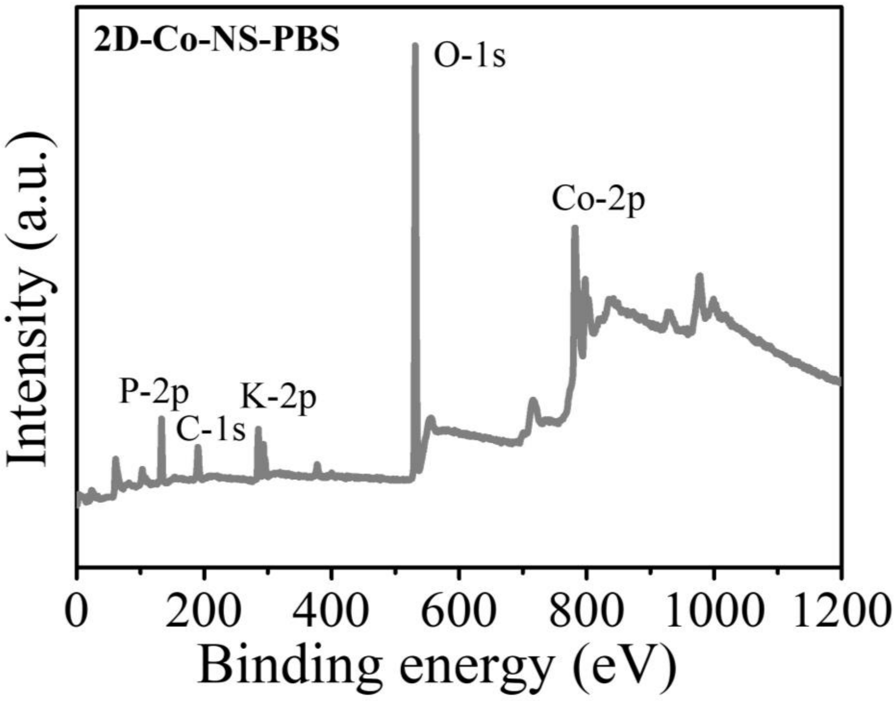

Figure S22. XPS spectrum of **2D-Co-NS-PBS**.

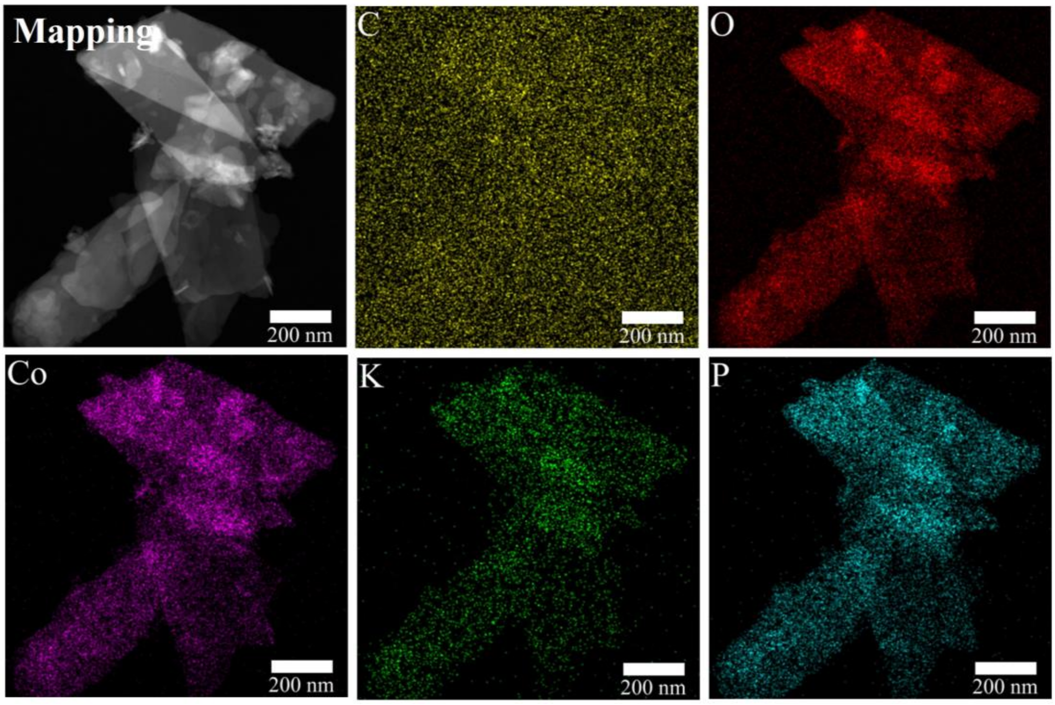

Figure S23. TEM-EDS mapping images of **2D-Co-NS-PBS**.

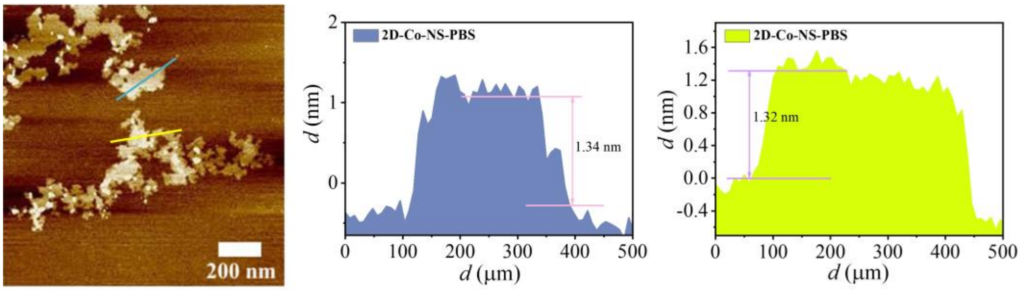

Figure S24. AFM images and height profiles of **2D-Co-NS-PBS**.

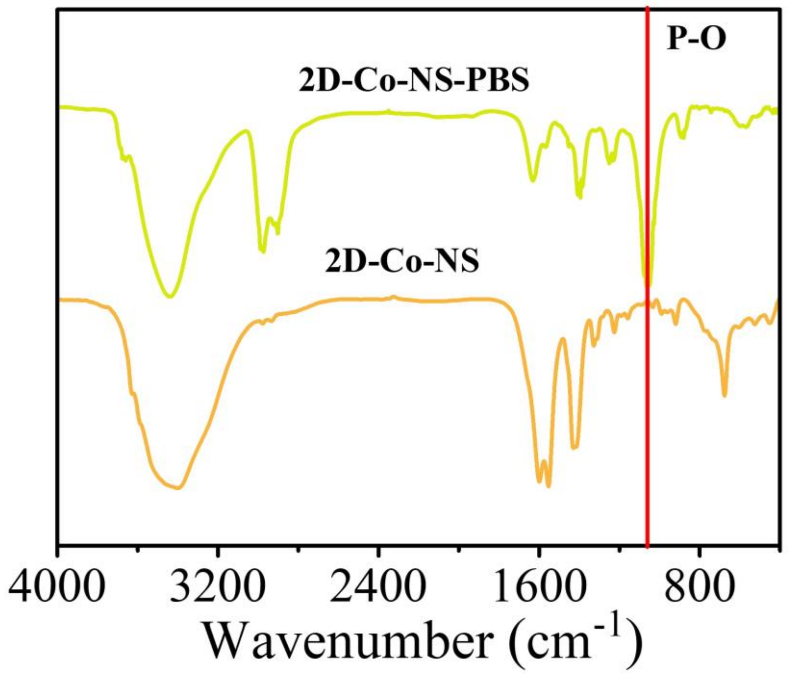

Figure S25. Infrared spectra of **2D-Co-NS** and **2D-Co-NS-PBS**.

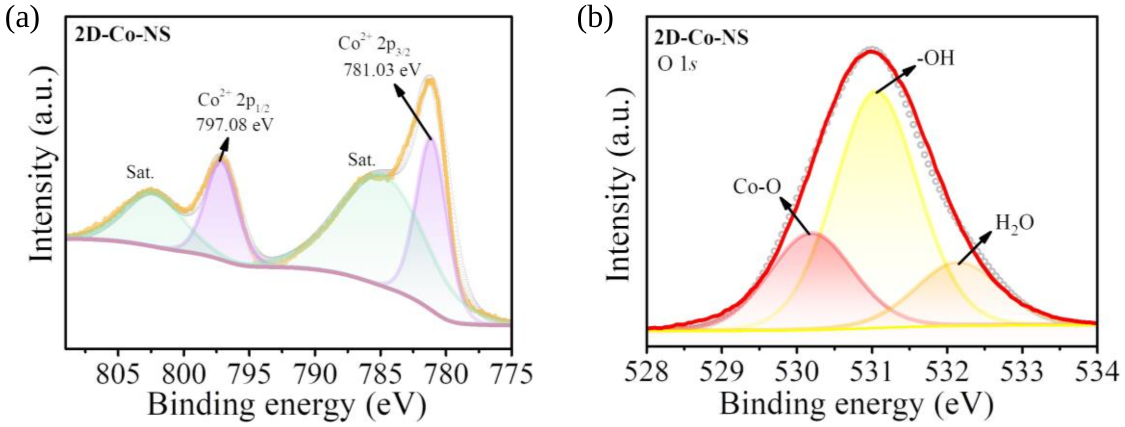

Figure S26. High-resolution XPS spectra of (a) Co2p and (b) O1s for **2D-Co-NS**.

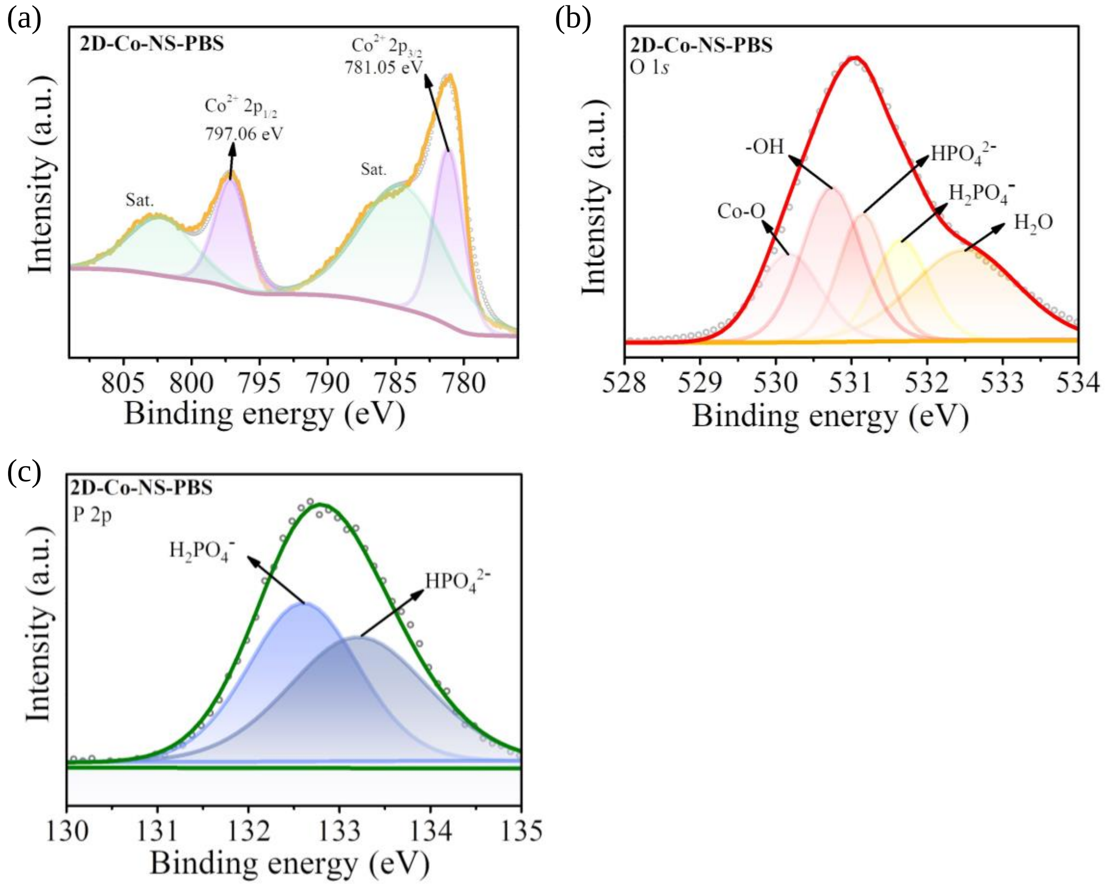

Figure S27. High-resolution XPS spectra of (a) Co2p, (b) O1s and (c) P2p for **2D-Co-NS-PBS**.

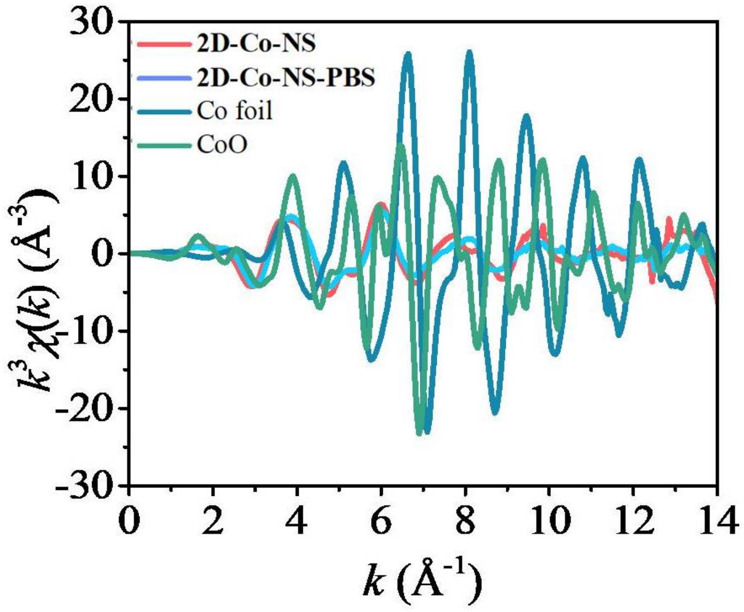

Figure S28. Fourier transform EXAFS spectra of Co-based samples.

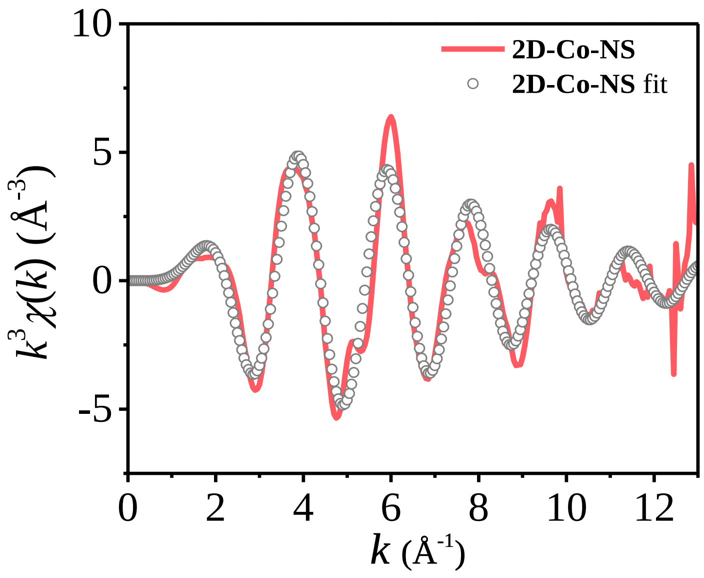

Figure S29. Fourier transform EXAFS spectra of Co-based samples.

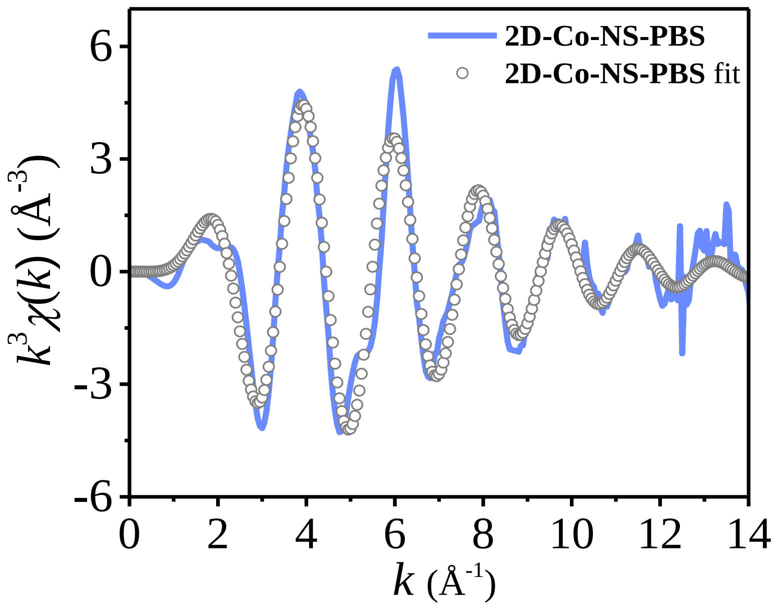

Figure S30. Fourier transform EXAFS spectra of Co-based samples.

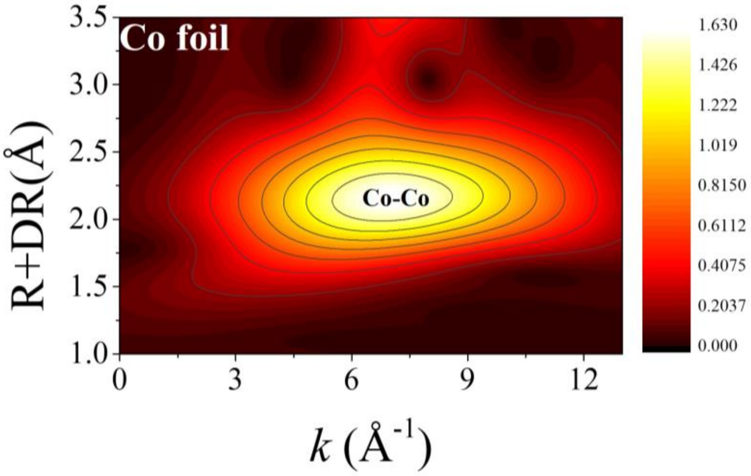

Figure S31. Results of wavelet Transform applied to EXAFS spectra of Co foil.

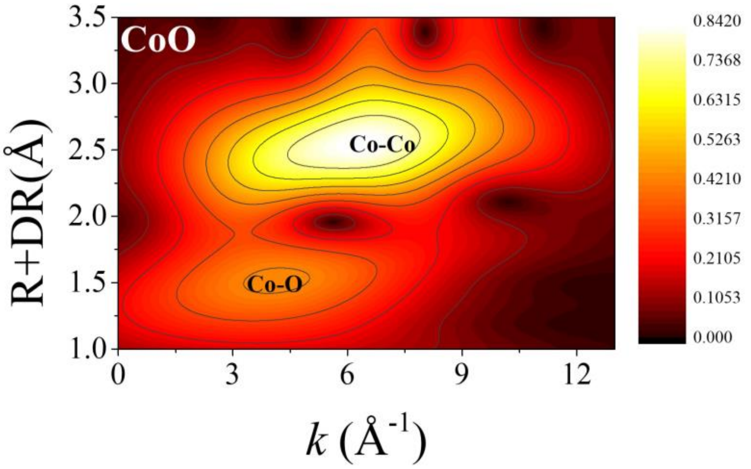

Figure S32. Results of wavelet Transform applied to EXAFS spectra of CoO.

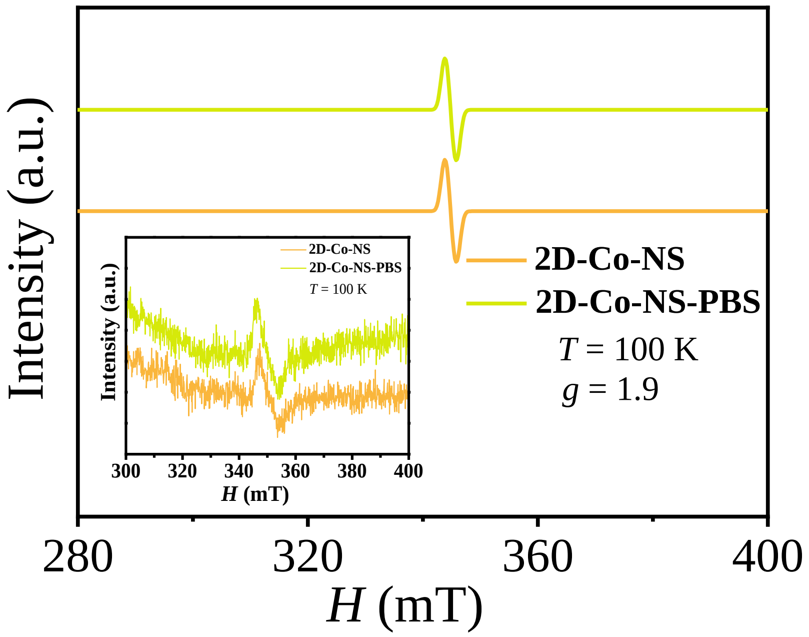

Figure S33. EPR spectrums of **2D-Co-NS** and **2D-Co-NS-PBS**.

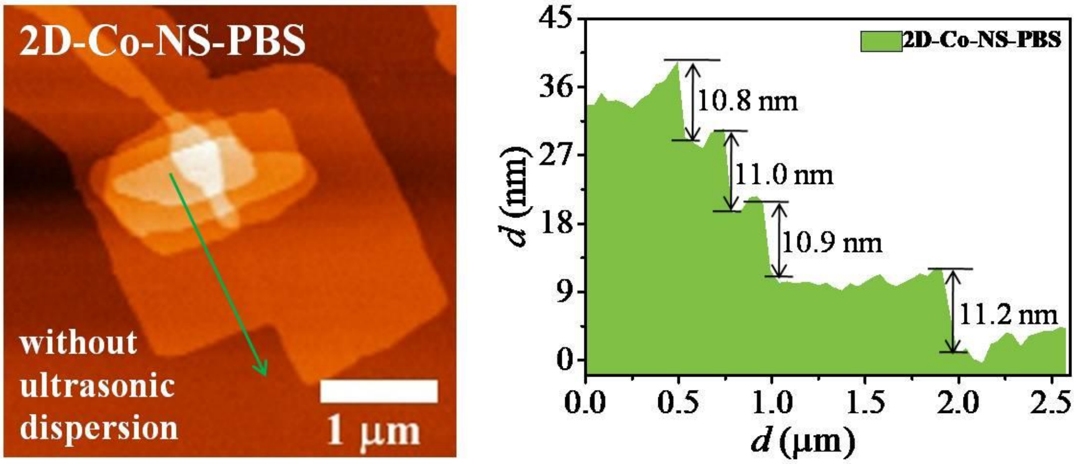

Figure S34. AFM images and height profiles of **2D-Co-NS-PBS** thick without ultrasonic dispersion.

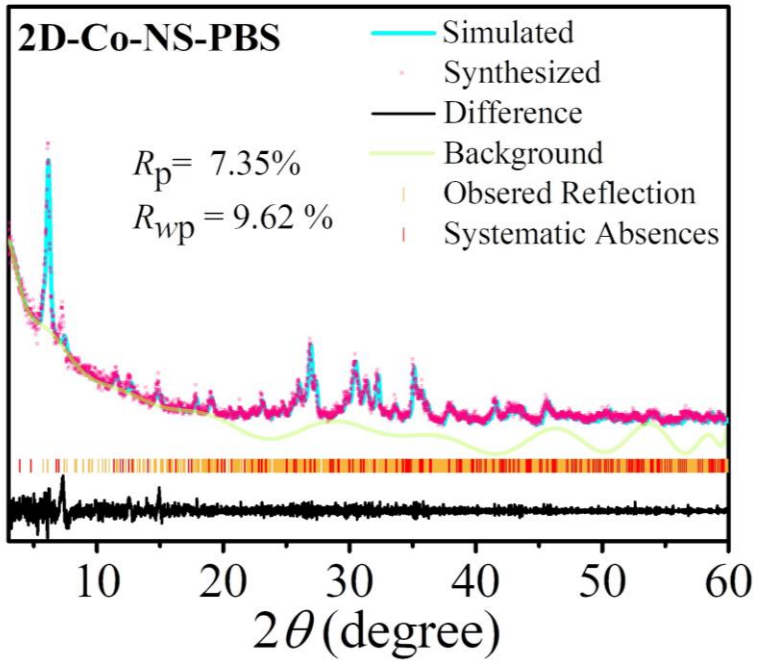

Figure S35. Final Rietveld refinement results of **2D-Co-NS-PBS**.

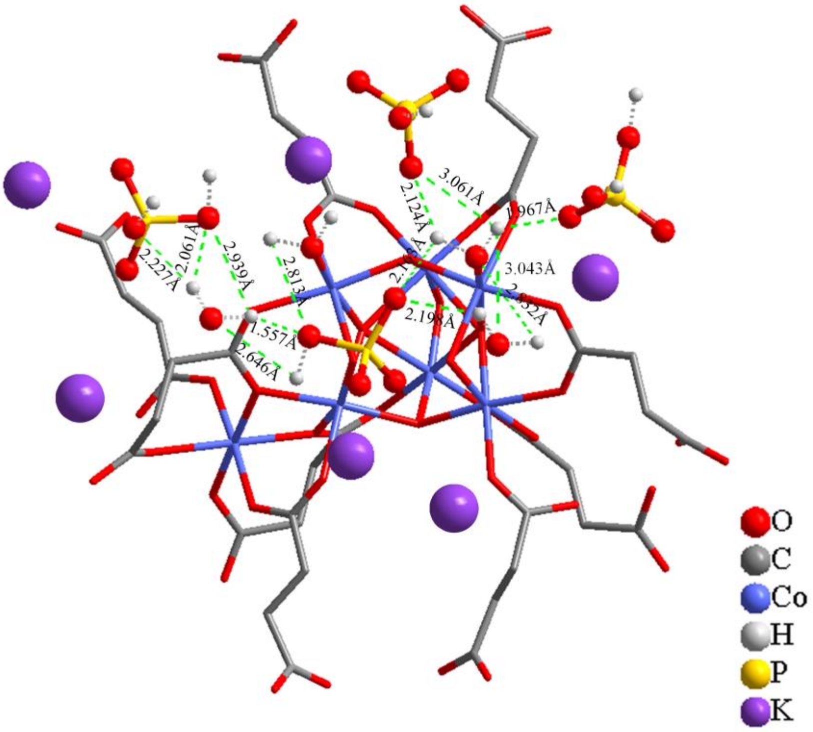

Figure S36. The asymmetric unit of **2D-Co-NS-PBS**. Dashed lines illustrates hydrogen bond.

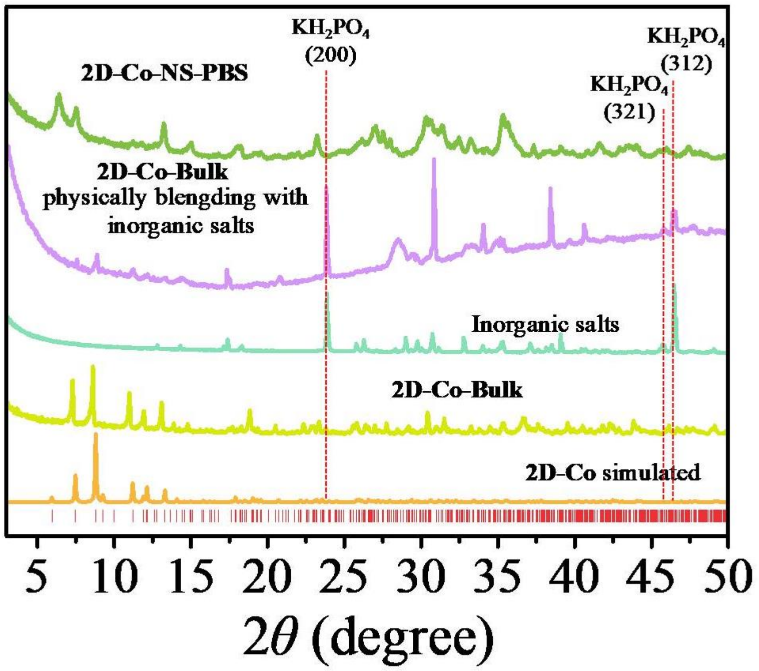

Figure S37. PXRD patterns of physical blending of **2D-Co-Bulk** and inorganic salts (the mixture of K_2HPO_4 and KH_2PO_4).

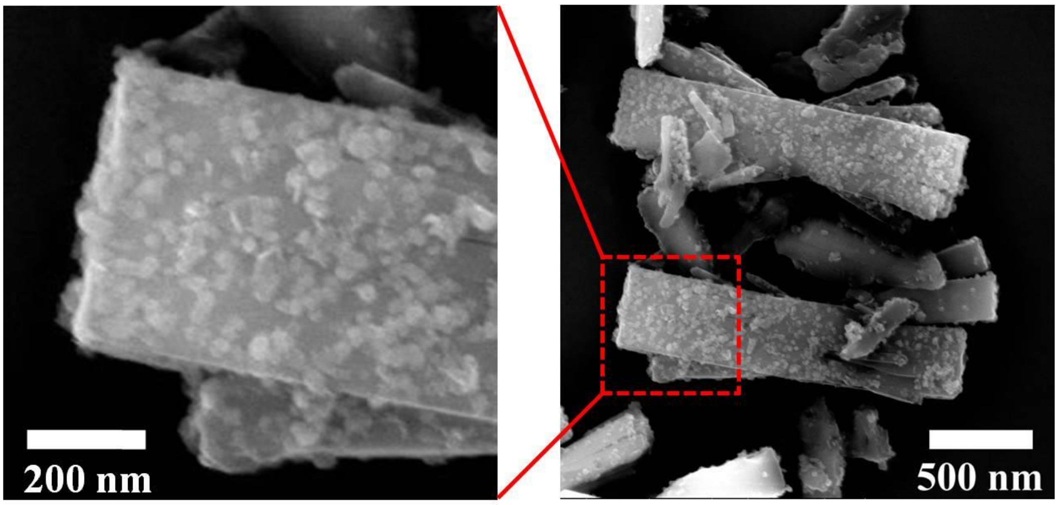

Figure S38. SEM image of physical blending of **2D-Co-Bulk** and inorganic salts (the mixture of K_2HPO_4 and KH_2PO_4).

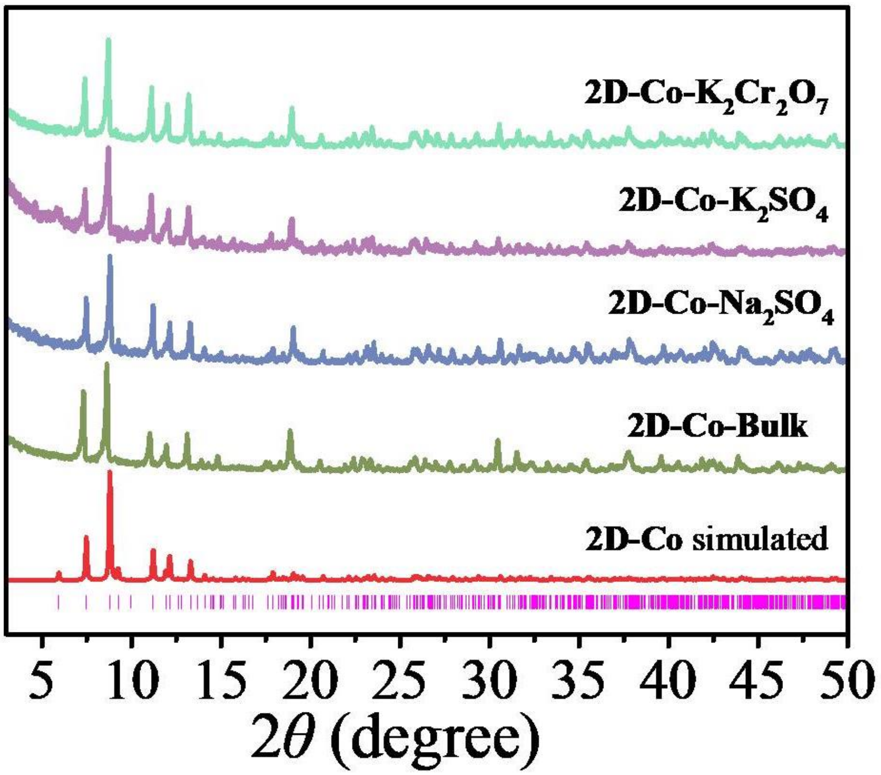

Figure S39. PXRD patterns of **2D-Co-Bulk** immersed in the different salts solutions.

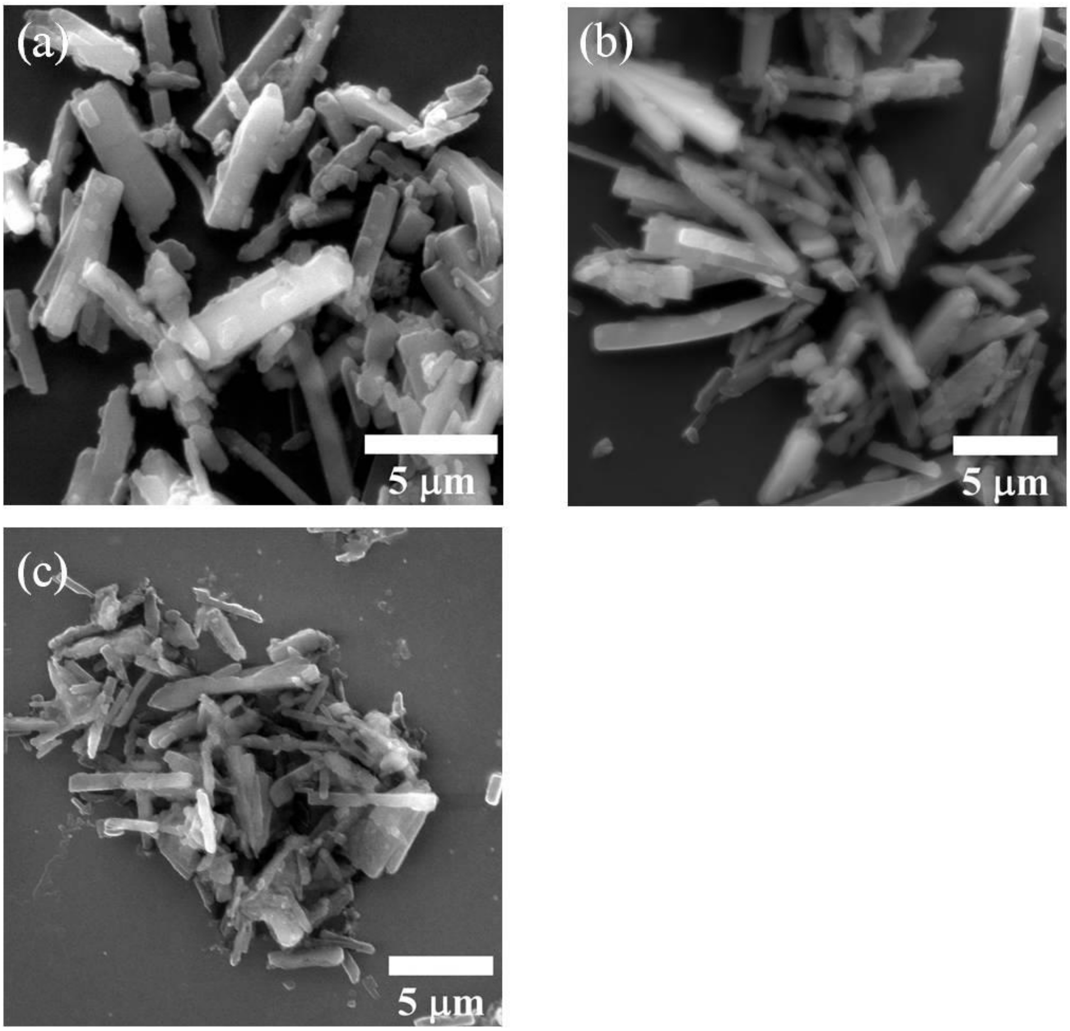

Figure S40. SEM images of **2D-Co-Bulk** immersed in (a) K_2SO_4 solution, (b) $K_2Cr_2O_7$ solution, and (c) Na_2SO_4 solution.

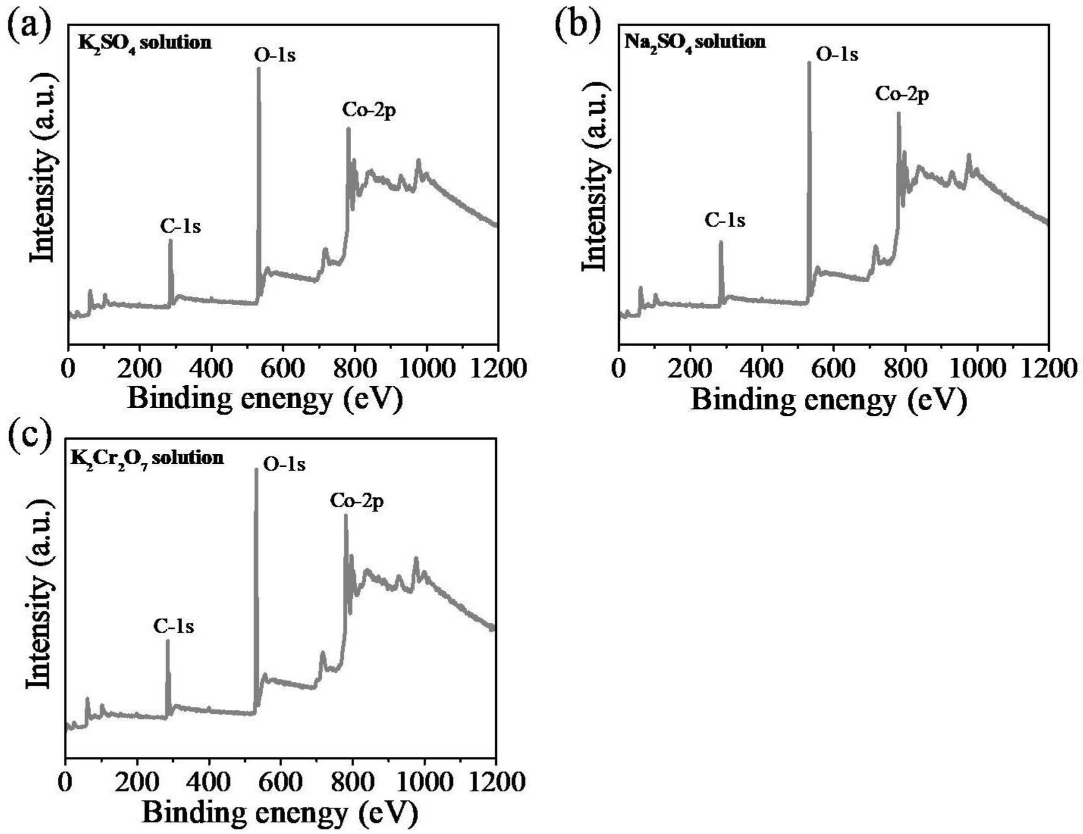

Figure S41. XPS spectrum of **2D-Co-Bulk** immersed in (a) K_2SO_4 solution, (b) Na_2SO_4 solution, and (c) $K_2Cr_2O_7$solution.

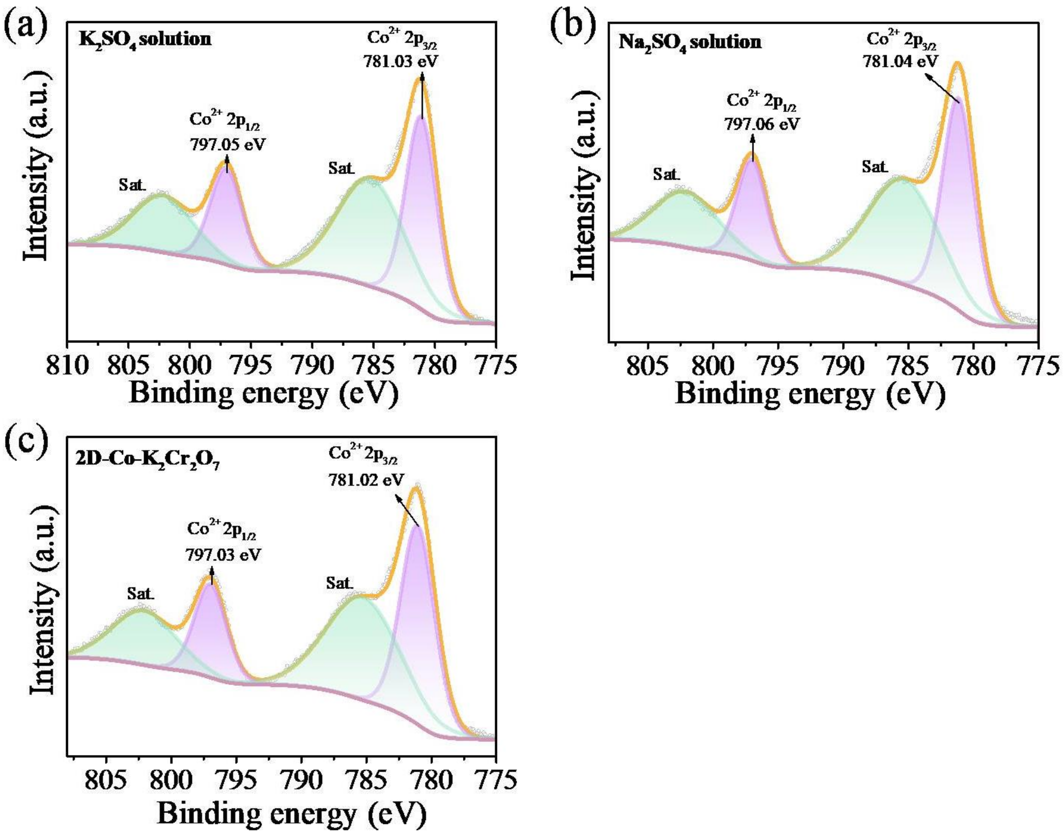

Figure S42. HR XPS spectra of Co2*p* of **2D-Co-Bulk** immersed in (a) K_2SO_4 solution, (b) Na_2SO_4 solution, and (c) $K_2Cr_2O_7$ solution.

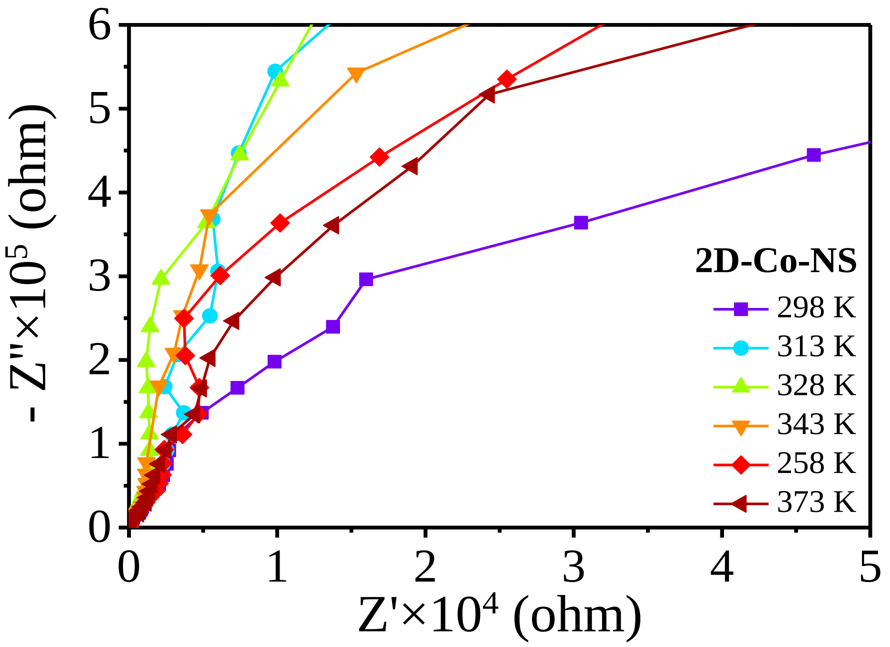

Figure S43. Nyquist plots with different temperature for **2D-Co-NS** under 99% RH.

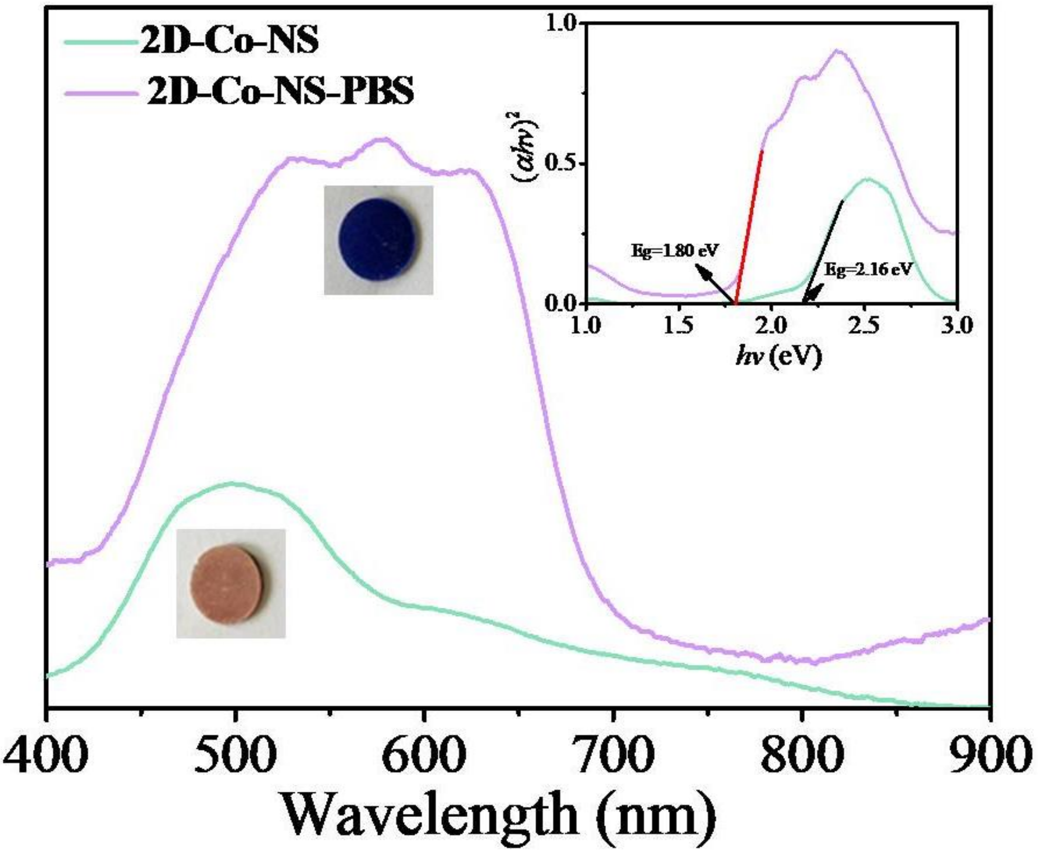

Figure S44. Diffuse reflectance UV−vis−NIR spectra of **2D-Co-NS** and **2D-Co-NS-PBS** exposed to ambient atmosphere for different lengths of time. Inset: Tauc plot of the data.

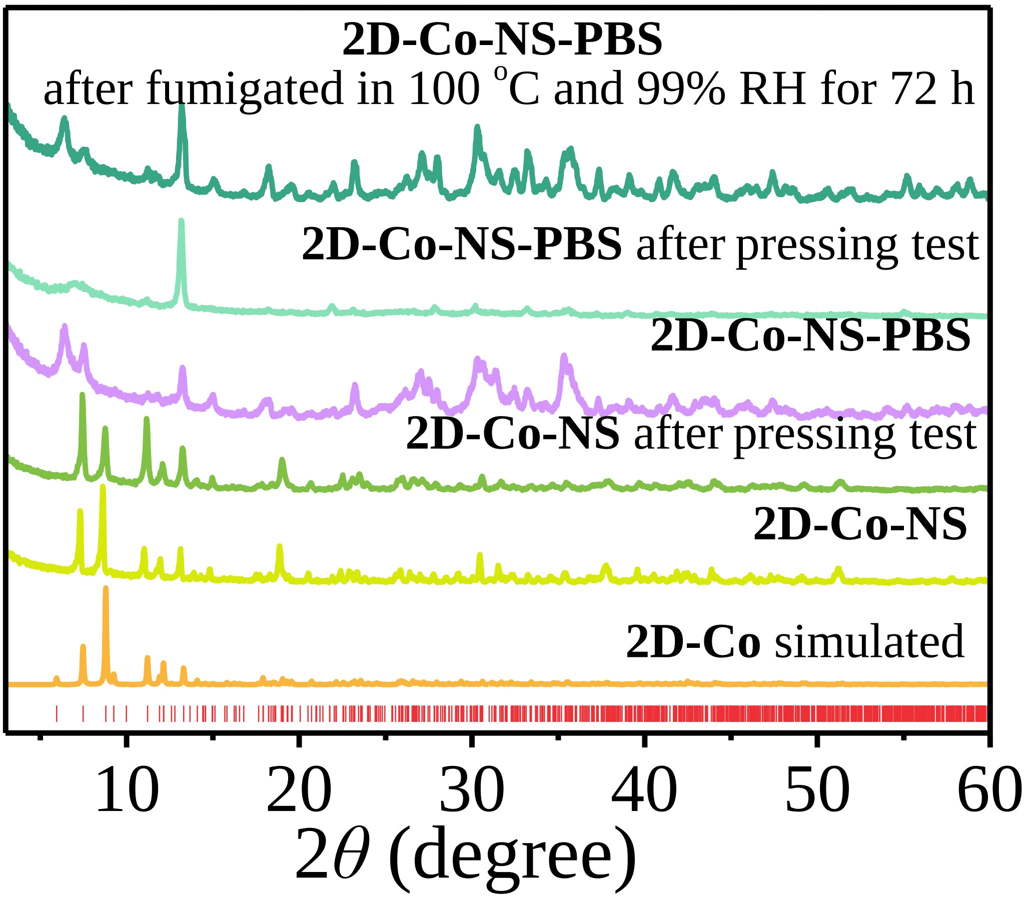

Figure S45. The PXRD pattern of **2D-Co-NS** pellets and **2D-Co-NS-PBS** pellets in different conditions.

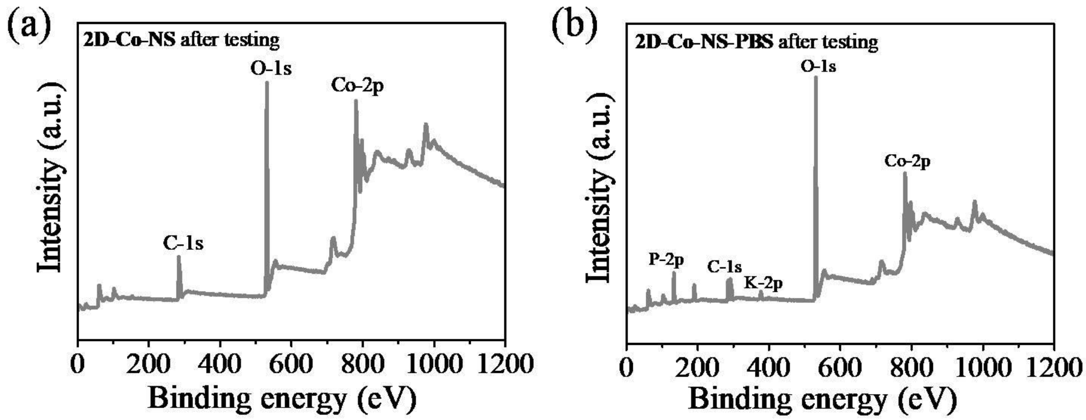

Figure S46. XPS spectrum of (a) **2D-Co-NS** pellets and (b) **2D-Co-NS-PBS** pellets after electrochemical testing.

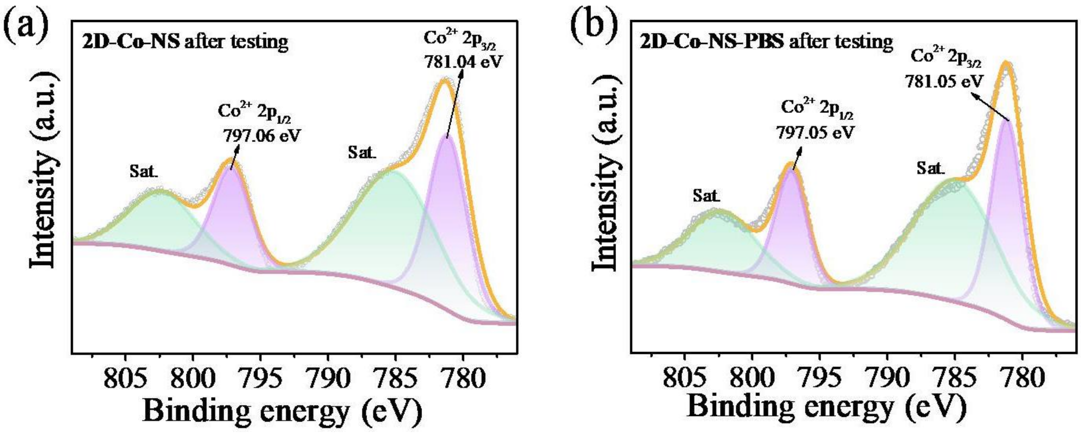

Figure S47. XPS spectrum of Co2p of (a) **2D-Co-NS** pellets and (b) **2D-Co-NS-PBS** pellets after electrochemical testing.

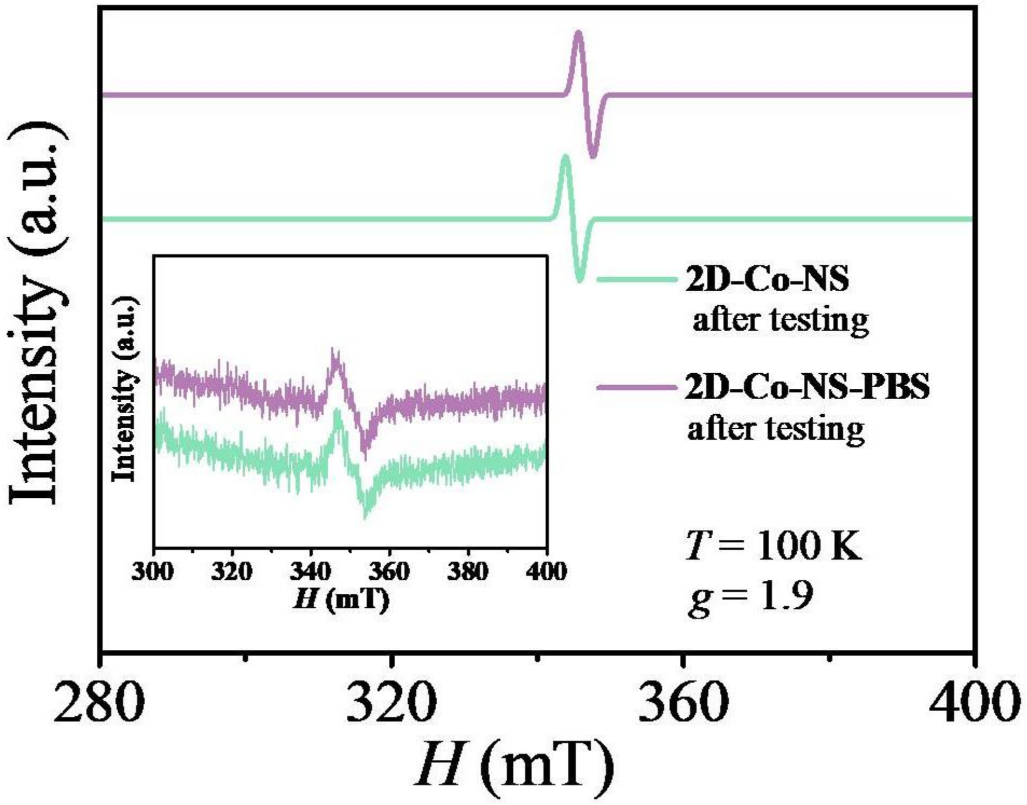

Figure S48. EPR spectrum of (a) **2D-Co-NS** pellets and (b) **2D-Co-NS-PBS** pellets after electrochemical testing.

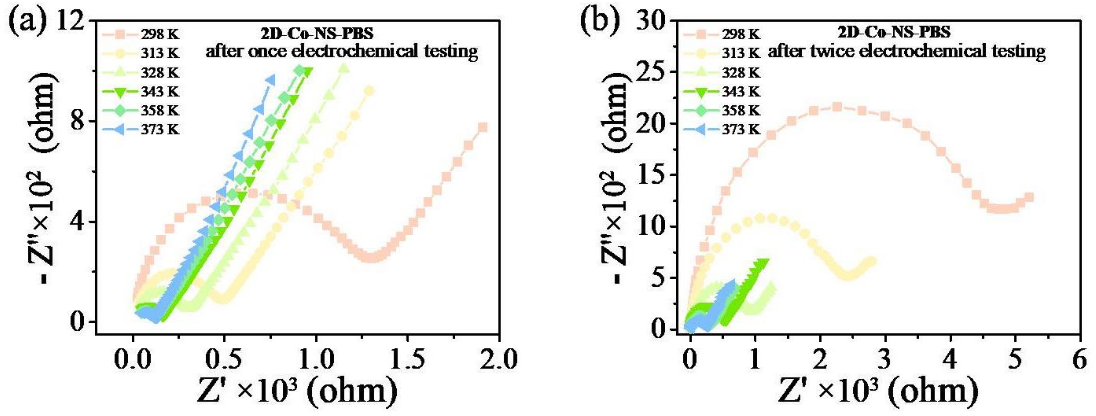

Figure S49. Nyquist plots with different temperature for **2D-Co-NS-PBS** under 99% RH after (a) once electrochemical testing and (b) twice electrochemical testing.

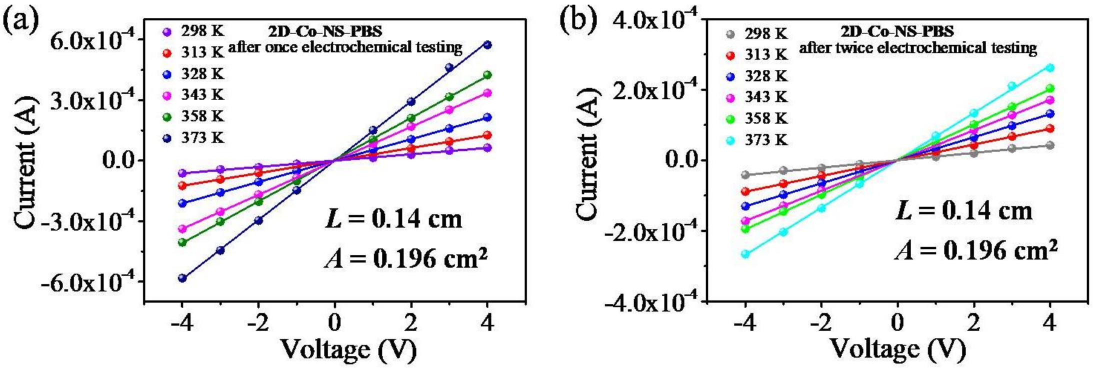

Figure S50. Representative *I-V* curves with different temperature for **2D-Co-NS-PBS** under 99% RH (a) once electrochemical testing and (b) twice electrochemical testing, the solid lines correspond to linear fits to the data.

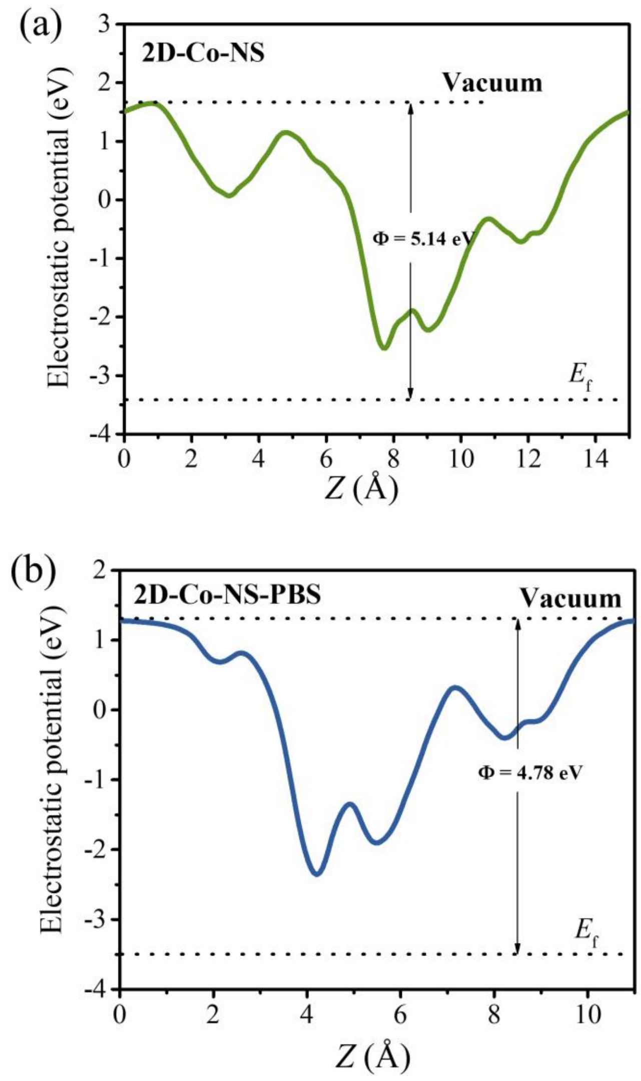

Figure S51. The work function (Φ) of **2D-Co-NS** and **2D-Co-NS-PBS**.

Table S1. The data of ICP-AES analysis

Sample	Co^{58}(mg/L)	K^{39}(mg/L)	Na^{23}(mg/L)	Cr^{52}(mg/L)	P^{30}(mg/L)	S^{32}(mg/L)
[a]**2D-Co-Bulk** immersed in 0.2 M PBS solution	0	54.6	0	0	288.3	0
[b]**2D-Co-Bulk**	4.43	0	0	0	0	0
[b]**2D-Co-NS-PBS**	4.40	1.86	0	0	0.421	0
[b]**2D-Co-Bulk** immersed in 0.2 M $K_2SO_{4(aq)}$	4.52	0	0	0	0	0
[b]**2D-Co-Bulk** immersed in 0.2 M $Na_2SO_{4(aq)}$	4.58	0	0	0	0	0
[b]**2D-Co-Bulk** immersed in 0.2 M $K_2Cr_2O_{7(aq)}$	4.35	0	0	0	0	0

a measurement and analysis with immersion solutionr; *b* measurement and analysis with the solid sample dissolved in 5% HNO_3 (5 mL) matrix and H_2O_2 (10 mL).

Table S2. EXAFS fitting parameters at the **Co** K-edge for various samples (S_0^2=0.792)

Sample	Shell	CN^a	R(Å)^b	σ^2(Å^2)^c	ΔE_0(eV)^d	R factor
Co foil	Co-Co	12*	2.49±0.01	0.0060±0.0005	7.6±0.7	0.0084
CoO	Co-O	6.0±0.2	2.13±0.01	0.0093±0.0008	1.1±1.0	0.0066
	Co-Co	12.0±0.3	3.00±0.02	0.0127±0.0020		
**2D-Co-NS**	Co-O	6.0±0.8	2.07±0.01	0.0069±0.0019	-0.9±1.2	0.0105
**2D-Co-NS-PBS**	Co-O	6.1±0.5	2.06±0.01	0.0091±0.0010	-0.7±0.9	0.0032

^{a}*CN*, coordination number; ^{b}*R*, distance between absorber and backscatter atoms; $^c\sigma^2$, Debye-Waller factor to account for both thermal and structural disorders; $^d\Delta E_0$, inner potential correction; *R* factor indicates the goodness of the fit. $S0^2$ was fixed to 0.792, according to the experimental EXAFS fit of Co foil by fixing *CN* as the known crystallographic value. Fitting range: 3.0 ≤ *k* (/Å) ≤ 12.6 and 1.0 ≤ *R* (Å) ≤ 3.0 (Co foil); 3.0 ≤ *k* (/Å) ≤ 11.4 and 1.0 ≤ *R* (Å) ≤ 3.5 (CoO); 2.0 ≤ *k* (/Å) ≤ 11.0 and 1.0 ≤ *R* (Å) ≤ 2.2 (52808Co-1 and 52808Co-2). A reasonable range of EXAFS fitting parameters: 0.700 < S_0^2 < 1.000; *CN* > 0; σ^2 > 0 Å^2; $|\Delta E_0|$ < 10 eV; *R* factor < 0.02.

Table S3. The crystallography parameters of **2D-Co** and the Rietveld refinements results of **2D-Co-NS-PBS**

Compound	**2D-Co**	**2D-Co-NS-PBS**
Chemical formula	$C_{16}H_{42}O_{32}Co_7$	/
Formula weight	1159.01	/
Temperature (K)	293(2)	/
Space group	$P2_1/c$	$P2_1/c$
a/Å	7.888(2)	15.687(8)
b/Å	19.082(6)	18.975(3)
c/Å	23.630(7)	23.495(5)
β/°	91.700(5)	91.697(3)
V/Å^3	3555 (2)	/
Z	4	/
D_c /g cm^{-3}	2.165	/
R_{int}	0.0642	/
R_1 (I>2σ)^a	0.0469	$R_p{}^c$ = 7.35%
wR_2 (all data)^b	0.136	$R_{wp}{}^d$ = 9.62%
Completeness	0.91	/
GOF	0.929	/

^{a} $R_1 = \Sigma||F_o| - |F_c||/\Sigma|F_o|$.

^{b} $wR_2 = [\Sigma w(F_o^2 - F_c^2)^2/\Sigma w(F_o^2)^2]^{1/2}$.

^{c} $R_p = \Sigma|cY^{sim}(2\theta_i) - I^{exp}(2\theta_i) + Y^{back}(2\theta_i)|/\Sigma|I^{exp}(2\theta_i)|$.

^{d} $R_{wp} = \{1/I^{exp}(2\theta_i)\,[cY^{sim}(2\theta_i) - I^{exp}(2\theta_i) + Yback(2\theta_i)]^2/\Sigma 1/I^{exp}(2\theta_i)[I^{exp}(2\theta_i)]^2\}^{1/2}$.

Table S4. Comparison of proton-electron dual-conductive performance for well-developed MPECs

Compounds	Condition	Proton conductivity (S/cm)	Electron conductivity (S/cm)	Reference
**2D-Co-NS**	100 °C, 99% RH	>>1.0×10^{-10}	2.1×10^{-8}	*This work*
**2D-Co-NS-PBS**		2.02×10^{-5}	3.8×10^{-4}	
Zn-HHTP-H_2O	70 °C, 98% RH	1.6×10^{-5}	4.5×10^{-2}	*10.1016/j.chempr.2022.09.016*
$(TTF)_2(1\text{-}H_6+\delta)$	25 °C, 40% RH	2.8×10^{-6}	1.3×10^{-4}	*J. Am. Chem. Soc.* **2022**, *144*, 17149
$[(CH_3)_2NH_2][In(TTFOC)]$	30 °C, 98% RH	1.3×10^{-2}	4.05×10^{-3}	*Matter*. **2020**, *2*, 711
$(H_3O)KCu_6Ge_2S_8\cdot nH_2O$	70 °C, 98% RH	2.49 × 10^{-2}	2.52 × 10^{-3}	*J. Phys. Chem. C* **2021**, *125*, 7034
$(H_3O)KCu_6Ge_2S_8\cdot nH_2O$	25 °C, 98% RH	1.99×10^{-3}	6.31×10^{-4}	*J. Phys. Chem. C.* **2021**, *125*, 7034
$h\text{-}WO_3\cdot nH_2O$	90 °C, 98% RH	3.7×10^{-3}	1.3×10^{-6}	*Nano Lett.* **2015**, *15*, 6802
Ag_xCS_2	167 °C, 98% RH	5.4×10^{-1}	2.12	*J. Electrochem. Soc*. **2001**, *148*, E237
